# Characterizing the Virome of Apple Orchards Affected by Rapid Decline in the Okanagan and Similkameen Valleys of British Columbia (Canada)

**DOI:** 10.3390/pathogens11111231

**Published:** 2022-10-25

**Authors:** Huogen Xiao, Wenjia Hao, Gavin Storoschuk, Jesse L. MacDonald, Hélène Sanfaçon

**Affiliations:** Summerland Research and Development Centre, Agriculture and Agri-Food Canada, Summerland, BC V0H 1Z0, Canada

**Keywords:** sudden apple decline, Rapid apple decline, apple ilarvirus 2, virus survey

## Abstract

Rapid apple decline disease (RAD) has been affecting orchards in the USA and Canada. Although the primary cause for RAD remains unknown, viruses may contribute to the incidence or severity of the disease. We examined the diversity and prevalence of viruses in orchards affected by RAD in the Okanagan and Similkameen Valleys (British Columbia, Canada). Next-generation sequencing identified 20 previously described plant viruses and one viroid, as well as a new ilarvirus, which we named apple ilarvirus 2 (AIV2). AIV2 was related to subgroup 2 ilarviruses (42–71% nucleotide sequence identity). RT-PCR assays of 148 individual leaf samples revealed frequent mixed infections, with up to eight viruses or viroid detected in a single tree. AIV2 was the most prevalent, detected in 64% of the samples. Other prevalent viruses included three ubiquitous viruses from the family *Betaflexiviridae* and citrus concave gum-associated virus. Apple rubbery wood virus 1 and 2 and apple luteovirus 1 were also readily detected. The thirteen most prevalent viruses/viroid were detected not only in trees displaying typical RAD symptoms, but also in asymptomatic trees. When compared with reports from orchards affected by RAD in Pennsylvania, New York State, and Washington State, regional differences in relative virus prevalence were noted.

## 1. Introduction

Rapid apple decline (RAD), also referred to as sudden apple decline, is a complex and devastating disease of commercial apple (*Malus domestica*) orchards first reported from northeastern and northwestern USA [1,2,3,4,5]. Estimated annual fresh apple production in Canada from 2018 to 2021 ranged from 382,640 to 440,713 tons with a farm gate value of 242,730,000 to 249,280,000 Canadian dollars (Statistics Canada, Area, production and farm gate value of marketed fruits available at https://www150.statcan.gc.ca/t1/tbl1/en/tv.action?pid=3210036401; accessed on 19 October 2022). The highest apple producing provinces are Ontario, Quebec, and British Columbia (BC) with apple production in BC representing close to 25% of the national production. RAD has been reported from these main apple producing provinces, as well as from Nova Scotia and Prince Edward Island [2,6,7]. In BC, the Okanagan and Similkameen valleys are major apple production areas. RAD symptoms in these regions include canker, peeling bark and necrosis around and above the rootstock-scion graft union, as well as small, yellowing, or desiccated leaves on the scion [8]. Severely affected trees eventually collapse. 

Although the primary cause of RAD remains undetermined, several factors may contribute to the disease, including pathogen pressure and abiotic stresses [3]. Apple trees are highly susceptible to virus infection, in part due to vegetative reproduction through grafting. Multiple viruses have been reported in apple trees worldwide, often occurring in mixed infection and these viruses have been associated with a variety of diseases [9]. Several viruses have also been detected in apple orchards affected by RAD in the USA often in mixed infection [1,3,4]. Although the presence of a specific virus or combination of viruses could not be clearly associated with RAD symptoms in the field [1,3,4], it has been suggested that viruses could contribute to the incidence or severity of the RAD in combination with other biotic or abiotic pressures [1,3,4]. Thus, evaluating the diversity of viruses in orchards affected by RAD remains of prime importance.

In Pennsylvania (PA), a next-generation sequencing (NGS) study using leaf samples collected from the scion and from the rootstock suckers of six diseased trees identified three common apple viruses from the family *Betaflexiviridae*: apple stem grooving virus (ASGV, genus *Capillovirus*), apple chlorotic leaf spot virus (ACLSV, genus *Trichovirus*), and apple stem pitting virus (ASPV, genus *Foveavirus*) [4]. These viruses are endemic in apple trees, frequently present in mixed infections and often referred to as latent viruses, due to the lack of specific symptoms associated with their presence, although symptomatology could vary with the virus strain and the apple scion and/or rootstock cultivar [9]. The PA study also identified a new luteovirus (apple luteovirus 1, ALV1), which was found at high prevalence in both diseased and asymptomatic trees [4]. Mixed infection of ALV1 with the three latent betaflexiviruses was common [4].

In New York State (NY), the prevalence of six known apple viruses was tested using DAS-ELISA and samples collected from roots and shoots of symptomatic and asymptomatic trees [3,10]. ASPV and ACLSV were the most prevalent and were detected in both symptomatic and asymptomatic trees [10]. ASGV, apple mosaic virus (ApMV, genus *Ilarvirus*, family *Bromoviridae*), and tomato ringspot virus (genus *Nepovirus*, family *Secoviridae*) were also detected although at lower incidence. Tobacco ringspot virus (also from the genus *Nepovirus*) was not detected [3,10].

In Washington State (WA), NGS of leaf and root tissues collected from four symptomatic trees and two asymptomatic trees revealed the presence of a large number of viruses, including some which had not been previously reported in apple [1,5]. Among the known viruses identified were ASPV, ACLSV, ASGV, and ApMV. Detection of apple green crinkle associated virus was also reported [1], although this virus is closely related to ASPV and may be considered a strain or variant of ASPV [11]. Other known plant viruses/viroids included apple hammerhead viroid (AHVd, genus *Pelamoviroid*, family *Avsunviroidae*), and three members of the family *Phenuiviridae*, apple rubbery wood virus 1 and 2 (ArWaV-1 and ArWaV-2, genus *Rubodvirus*), and citrus concave gum-associated virus (CCGaV, genus *Coguvirus*). CCGaV was detected for the first time in apple trees [1,5]. Of the known viruses/viroid, ASPV, ASGV, and ApMV were the most abundant. Other prevalent viruses included ACLSV, ArWaV2, AHVd, and CCGaV. Seventeen new viruses were also detected by NGS [1]. Many of these viruses showed strongest homology to fungal or insect viruses. Other putative new viruses were related to known plant viruses, including apple ilarvirus 1 (AIV1) and two apple tombus-like viruses. 

The studies above highlight the diversity of viruses found in apple orchards in the USA. They also highlight regional differences in virus incidence. For example, ALV1, which was detected at high prevalence in PA [4], was not detected in WA [1]. The incidence and diversity of viruses present in apple orchards affected by RAD have not been studied in BC. This study aimed to address this gap in knowledge. We used NGS as an unbiased method to detect both known apple viruses as well as any emerging new viruses [12,13]. Previous studies have shown that plant viruses infecting apple are readily detected in leaf samples [1,4,10]. To evaluate the diversity of viruses present in BC, we combined leaf samples collected from nine conventionally managed apple orchards in the Okanagan Valley (BC), and one organic orchard in the Similkameen Valley (BC). The combined samples were also selected to represent the range of observed symptoms, scion/rootstock cultivars, geographic locations, and sampling times. Downstream RT-PCR assays on individual leaf samples were used to assess the relative incidence of the most prevalent viruses/viroids identified by NGS. The results highlight both similarities and differences in the relative incidence of viruses in affected apple orchards from BC and from various apple-producing areas in the USA.

## 2. Results

### 2.1. Detection of 21 Plant Viruses and One Plant Viroid by NGS in Apple Orchards Affected by RAD

Initial apple orchard surveys in the Okanagan Valley in 2018 revealed tree mortality of up to 40% in extreme cases, as estimated by visual inspection of selected orchards. One orchard was so sparse with trees that it was completely removed after sampling. From the responses to a questionnaire sent to growers, rapid decline symptoms were noted throughout the Okanagan and Similkameen Valleys from as far south as Osoyoos, BC and north to Vernon, BC, suggesting that it is a widespread issue encompassing the majority of the apple growing regions in BC. To obtain a snapshot of plant viruses present in orchards affected by RAD in the Okanagan and Similkameen valleys of British Columbia (Canada), we collected 148 individual leaf samples from nine experimental and commercial conventionally managed orchards in Summerland in the South Okanagan Valley and from one commercial organic orchard in Cawston in the Similkameen Valley. The samples represented different types of rootstocks/scions cultivar and were collected from diseased trees as well as asymptomatic trees (Table 1, see Material and Methods for more details). Next, we produced ten composite leaf samples for NGS. Each composite sample was a mixture of three selected individual leaf samples (Table 2). This strategy allowed us to limit the number of samples sent for NGS sequencing, while ensuring that the selected samples represented most cultivars, orchard locations, sampling times, and types of symptoms.

A total of 71–103 million reads were obtained for each composite sample. Mapping to the reference genome of *Malus domestica* (GDDH13v1.1) to eliminate host sequences resulted in 5.5–9.8 million unmapped reads. *De novo* assembly from these unmapped reads generated 2937 to 10,535 contigs with length varying from 250 to 11,148 nucleotides (nts). Blast search and remapping of these contigs against a database of plant virus sequences revealed the presence of 21 viruses and one viroid (AHVd) in the ten NGS samples (Table 3 and Table 4). Twenty of these viruses and the viroid were previously described. In addition, an ilarvirus sequence was readily detected that was related to previously described plant ilarviruses, but clearly distinct. Other viruses were also detected that showed sequence identities to known fungal or insect viruses but are not reported here.

The viroid AHVd was detected in seven composite samples with 100% genome sequence coverage. The highest number of reads mapping to AHVd was 180,962 in sample HX8 (Table 4 and Table S1), which was collected from three trees that did not show typical RAD symptoms (Table 2). 

The ubiquitous ASPV, ACLSV, and ASGV from the family *Betaflexiviridae* were readily detected in our composite samples (Table 4 and Table S1). ASPV was detected in eight composite samples, with genome coverage ranging from 6.3% to 99%. Variability in ASPV sequences was very high, with nt sequence identity to the reference isolate ranging from 80% to 99%, suggesting the presence of multiple divergent variants in each composite sample. ACLSV and ASGV were found in six and three composite samples, respectively. Genome coverage ranged from 86% to 96% and percentage of sequence identity to the reference isolates ranged from 82% to 99%, again suggesting high diversity of sequence in the BC isolates (Table S1). Because multiple variants of ASPV, ACLSV, and ASGV were detected in each composite samples, assembly of near-complete genome sequence of individual variants proved difficult and was not investigated further. Cherry virus A (CVA), another member of the family *Betaflexiviridae*, was detected at low incidence in only one composite sample (187 reads mapped to CVA in sample HX1, Table 4 and Table S1).

**Figure 1:**
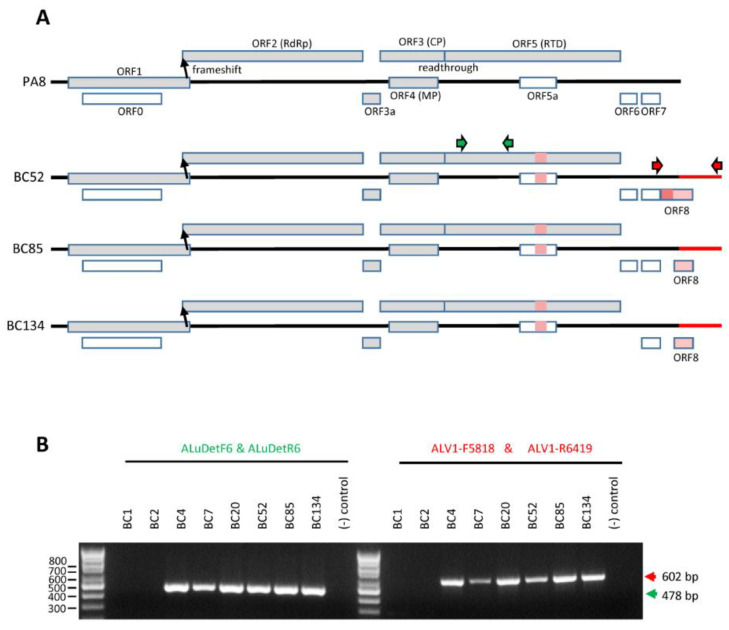
Genome structure of BC isolates of ALV1 compared to that of the reference PA8 isolate. (**A**) Schematic representation of the ALV1 genomic RNA. Open reading frames are shown as rectangular boxes and the 5′ and 3′ UTRs are represented with lines. The predicted function of each putative protein is indicated, when known. The biological function (if any) of ORFs shown in white is not known. Areas highlighted in red or pink indicate significant differences between the BC and PA8 isolates, including the presence of small in-frame deletions in ORF5 and ORF5a, and a 3′ UTR extension in BC isolates, which includes conserved small ORF8. The position of primers used for RT-PCR detection in panel B is shown, including previously described primers ALuDetF6 and ALuDetR6 (in green) [4] for general detection of ALV1 and primers ALV1-F5818 and ALV1-R6419 (in red) for detection of the 3′ UTR extension in the BC isolates (see Table S2 for primer sequences). (**B**) RT-PCR detection of ALV1 in individual samples. On the left of the gel, a previously described standard set of primers was used to confirm the presence of ALV1 in individual samples BC4, BC7, BC20, BC52, BC85, and BC134. BC1 and BC2 were not infected by ALV1. A negative control (no RNA added) was also included. On the right, a specific primer set was used to confirm the presence of the 3′ UTR extension in BC isolates that tested positive for ALV1. Molecular mass markers (in base pairs) are labelled on the left of the gel. The expected size of the RT-PCR products is indicated on the right of the gel. RdRp, RNA-dependent RNA polymerase; CP, coat protein; RTD, readthrough domain; MP, movement protein.

The luteovirus ALV1 was detected in five composite samples with reads ranging from 1978 for sample HX4 to 39,292 for HX1. Nucleotide sequence identity to the reference ALV1 genome from Pennsylvania (PA8) isolate (NC_040680.1) ranged from 94% to 99% (Table 4 and Table S1), and genome coverage was above 100%, due to the longer genomic RNAs of BC isolates (see below). We selected three composite NGS samples, of which each was later found to include only one ALV1 positive tree and assembled near full-length genome contigs. The selected samples were HX4 (including ALV1-positive individual sample BC52), HX7 (including ALV1-positive individual sample BC85), and HX10 (including ALV1-positive individual sample BC134). Sequences have been deposited in the NCBI database with accession numbers OP271661, OP271662, and OP271663 for contigs corresponding to individual samples BC52, BC85, and BC134, respectively (annotated sequence are also available as Text S1 to S3). Interestingly, the length of the contigs assembled ranged from 6362 to 6390 nts, whereas the complete genomic RNA length for the reference PA8 isolate was reported to be 6001 nts [4]. An extension of at least 420 nts at the 3′ end of the ALV1 RNA 3′ untranslated region (UTR) was noted in all sequenced samples (Figure 1A). Presence of this 3′ UTR extension in the BC isolates was confirmed by RT-PCR (Figure 1B). The general genomic RNA organization of ALV1 BC isolates was similar to that described for the PA8 isolate with a few minor differences (Figure 1A). All major ORFs were highly conserved between the BC and PA8 isolates including ORF0, which was already noted to have a sequence unique to ALV1 with no apparent sequence homologies with the corresponding ORF0 of other luteoviruses [4]. Two small in-frame deletions were noted in the ORF5 of the BC isolates when compared to the PA8 isolate (Figure 1A, Figure S1). This region of ORF5 overlaps with small ORF5a and similar small deletions were also noted in ORF5a of the BC isolates (Figure 1A, Figure S1). Small ORFs 6 and 7, which were noted previously in the PA isolate, were also detected in most BC isolates, although ORF6 was absent from the BC134 contig (Figure 1A). Finally, an additional small ORF was detected in the 3′ UTR extension of the BC isolates (ORF8, Figure 1A). Although ORF8 was slightly larger in the BC52 contig due to a mutation of an in-frame stop codon upstream of the ORF, the C-terminal sequence of ORF8 was highly conserved among the BC isolates (Figure S1). It is not known whether small ORFs 5a, 6, 7, or 8 are expressed in infected plants.

CCGaV, ARWaV-1, and ARWaV-2 from the family *Phenuiviridae* were readily detected in three to four composite samples each with nt sequence identities of 99.3–99.9%, 93–99%, and 95–99.8% to the reference isolates, respectively (Table 4 and Table S1).

Numerous ilarviruses have been reported to infect fruit trees, including apple trees [14]. Six known ilarviruses were detected in low abundance and incidence (Table 4 and Table S1). ApMV, tobacco streak virus (TSV), and prunus necrotic ringspot virus (PNRSV) were only found in one composite sample. The partially sequenced AIV1, first identified in WA [1], was found in two composite samples with only low read numbers. Solanum nigrum ilarvirus (SNIV) was detected in six composite samples, but also showed a low number of reads.

Several contigs from each of the ten composite samples showed around 90% nt sequence identity to the partially sequenced blacklegged tick-associated ilarvirus (BLTaIV), a poorly described virus discovered in an NGS study of environmental tick samples [15]. The contigs were also related to known plant ilarviruses, although with only 52–71% nt sequence identity with the RNA1, RNA2, and RNA3 of the closest relative, citrus variegation virus (CVV). Further characterization of this virus, which we named apple ilarvirus 2 (AIV2), is presented below.

Only low read numbers were noted for other plant viruses including clover yellow mosaic virus and white clover mosaic virus (genus *Potexvirus*), detected in five and four composite samples, respectively; turnip vein clearing virus (genus *Tobamovirus*) and turnip mosaic virus (genus *Potyvirus*) each detected in three composite samples; as well as turnip crinkle virus (genus *Betacarmovirus*) and sowbane mosaic virus (genus *Sobemovirus*) each detected in only one composite sample (Table 4 and Table S1).

### 2.2. Virus Prevalence in Apple Orchards Affected by RAD 

RT-PCR was conducted on the 148 individual leaf samples (each corresponding to a single tree) to determine the field prevalence of the twelve viruses and one viroid for which there were more than 200 reads in the composite NGS samples (i.e., AHVd, ACLSV, ASGV, ASPV, AIV2, ApMV, SNIV, TSV, PNRSV, ALV1, ARWaV-1, ARWaV-2, and CCGaV, see Table S2 for primer sequences). Of the 148 individual samples tested, 147 trees were found to be infected with at least one of the tested viruses (Table 5). The majority of the trees were infected by more than two of the tested viruses (81.7% of the individual samples), with up to eight viruses and/or viroid detected in a tree (Table 5). Thus, mixed infections were very common in the orchards.

Virus detection rates ranged from 63.5% of the individual samples for AIV2 to 0.7% for PNRSV (Table 6). The most prevalent viruses were AIV2 (63.5%), CCGaV (61.2%), ASPV (54.7%), ACLSV (50%), and ASGV (41.9%). The last three viruses are known as latent viruses in apples and are widely detected worldwide. Thus, it was not surprising to find them in high abundance in the collected samples. CCGaV was initially associated with concave gum disease in citrus but was recently detected at high prevalence in apple trees in Washington State, USA [1,5]. In contrast, AIV2, which was detected with the highest overall field prevalence, has not been previously reported in association with apple trees. Other ilarviruses were only detected at low incidence. TSV, SNIV3, and ApMV were detected in 9.5%, 7.4%, and 4.1% of the individual samples, respectively, while PNRSV was only found in one sample. The viroid AHVd was detected in 23% of the samples. Three viruses newly identified in apple orchards elsewhere, ALV1 (first detected in PA) as well as ARWaV1 and ARWaV2 (both first reported from WA), were readily detected in orchards from the Okanagan and Similkameen Valleys of British Columbia. Indeed, these three viruses were found in 18.2%, 8.8%, and 15.5% of the samples, respectively (Table 6).

We compared virus incidence in samples collected from trees with RAD symptoms to that in samples collected from non-symptomatic trees (Table 6). There was no clear association of a single virus with the presence of typical RAD symptoms, although the incidence of ALV1, ASGV, ARWaV1, SNIV3, ARWaV2, and ApMV was slightly higher (1.2–4.8%) in samples collected from diseased trees. In contrast, the incidence of CCGaV, ASPV, TSV, AHVd, ACLSV, and AIV2 was slightly lower (1.9–13.1%) in these samples. As stated above, mixed infection was common. However, we could not find a clear association between a specific combination of viruses (or viroid) and the presence of RAD symptoms.

### 2.3. Complete Genome Sequence and Phylogenetic Analysis of Apple Ilarvirus 2

AIV2, which was identified in our NGS dataset, was the most prevalent virus in our individual samples. Thus, we decided to determine the complete genomic sequence of the virus and examine its phylogenetic relationship with other ilarviruses.

**Figure 2:**
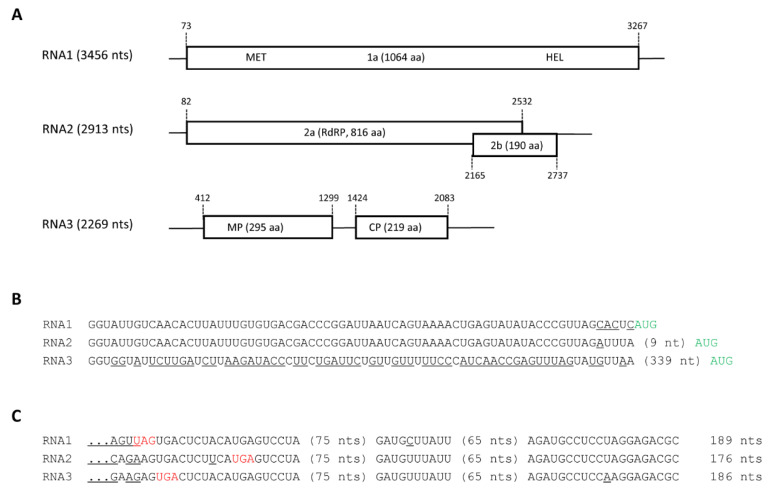
Genome structure of AIV2. (**A**) Schematic representation of the three AIV2 RNAs. Open reading frames are shown as rectangular boxes and the 5′ and 3′ UTRs are illustrated with lines. The length of each RNA is indicated in parentheses. The nt position of the start and end of each ORF as well as the size and predicted function of each putative protein are indicated. (**B**) The sequence of the 5′ UTRs of the three RNA segments are shown with mismatched nts underlined. In the 5′ UTRs of RNA2 and RNA3, sequences of 9 nts and 339 nts immediately upstream of the AUG start codon (highlighted in green) are not shown. (**C**) The sequence of the 3′ UTRs of the three RNA segments are shown with mismatched nts underlined. Two regions of 75 and 65 nts with 100% sequence identity amongst the three RNAs are not shown. Stop codons are highlighted in red. The length of the 3′ UTR is indicated on the right. MET, methyltransferase; HEL, helicase; RdRp, RNA-dependent RNA polymerase, MP, movement protein; CP, coat protein.

The complete sequence of the three AIV2 RNAs was obtained by compilation of the NGS datasets, 5′ and 3′ RACE as well as Sanger sequencing of at least three individual RT-PCR clones for each RNA (see Material and Methods). The sequence was deposited in the NCBI database under accession numbers ON932434 to ON932436 (annotated sequence is also available as Text S4). The genomic organization of the RNAs is similar to that of subgroups 1 and 2 ilarviruses [16] (Figure 2A). RNA1 contains a single ORF that encodes protein 1a (a putative replicase) of 1064 amino acids (aa) (120.44 kDa). Typical methyltranferase (aa location 59–405) and helicase (aa location 781–1032) domains were identified in this protein. RNA2 has two ORFs overlapping by 368 nts, coding for protein 2a of 816 aa (92.95 kDa), which has canonical RNA-dependent-RNA-polymerase motifs (aa 234–649) and protein 2b of 190 aa (21.73 kDa). Protein 2b is only found in ilarviruses of subgroups 1 and 2 and may function in viral movement and as a suppressor of RNA silencing [16,17]. RNA3 contains two non-overlapping ORFs, coding for a movement protein of 295 aa (33.12 kDa) and a coat protein of 219 aa (23.98 kDa). The first three nts in the 5′ UTRs were identical for all three RNAs and the first 67 nts in the 5′ UTR were identical for RNA1 and RNA2 (Figure 2B). The last 191 nts of the 3′ end (including the 3′UTR) were almost identical (with only three mismatches) for all three RNAs (Figure 2C). 

**Figure 3:**
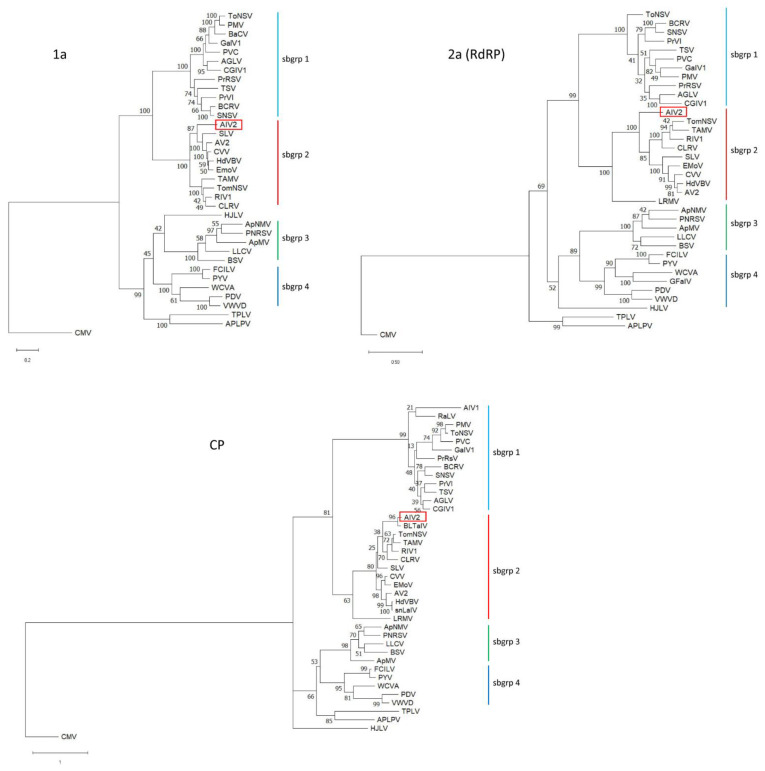
Phylogenetic relationships between AIV2 and other ilarviruses. Complete or near-complete deduced amino acid sequences for the 1a, 2a (RdRp), and coat protein (CP) were aligned using Clustal W as implemented in MegaX. Phylogenies were generated using the maximum likelihood method. Cucumber mosaic virus (CMV, a cucumovirus) was used as an outgroup to root the trees. The results of bootstrap analyses (1000 replicates) are shown for each branch. Virus acronyms are defined as follows: AGLV (ageratum latent virus), APLPV (American plum line pattern virus), AIV1 (apple ilarvirus 1), AIV2 (apple ilarvirus 2), ApMV (apple mosaic virus), ApNMV (apple necrotic mosaic virus), AV2 (asparagus virus 2), BaCV (bacopa chlorosis virus), BCRV (blackberry chlorotic ringspot virus), BLTaIV (blacklegged tick associated ilarvirus), BSV (blueberry shock virus), CGIV1 (Cape gooseberry ilarvirus 1), CLRV (citrus leaf rugose virus), CVV (citrus variegation virus), EMoV (elm mottle virus), FCILV (Fragaria chiloensis latent virus), GaIV1 (grapevine associated ilarvirus 1), GFaIV (Gungahlin flea-associated ilarvirus), HJLV (Humulus japonicus latent virus), HdVBV (hydrangea vein banding virus), LLCV (lilac leaf chlorosis virus), LRMV (lilac ring mottle virus), PMV (parietaria mottle virus), PVC (peanut virus C), PYV (potato yellowing virus), PrRSV (privet ringspot virus), PDV (prune dwarf virus), PNRSV (Prunus necrotic ringspot virus), PrVI (Prunus virus I), RaLV (Raphanus latent virus), RIV1 (Rosa ilarvirus 1), SLV (spinach latent virus), SNSV strawberry necrotic shock virus), snLaIV (surrounding non-legume associated ilarvirus), TPLV (tea plant line pattern virus), TSV (tobacco streak virus), ToNSV (tomato necrotic spot virus), TomNSV (tomato necrotic streak virus), TAMV (Tulare apple mosaic virus), VWVD (Viola white distortion associated virus), WCVA (water chestnut virus A). See Table S3 for the list of accession numbers.

To investigate the relationships between AIV2 and other ilarviruses, phylogenetic trees were generated based on alignments of the deduced amino acid sequence of proteins 1a, 2a, and CP. AIV2 clustered with subgroup 2 ilarviruses, but was clearly distinct from other plant ilarviruses (Figure 3). Nucleotide sequence identities with subgroup 2 ilarviruses were between 50 and 71% for RNA1, 49 and 67% for RNA2, and 42 and 57% for RNA3. As mentioned above, AIV2 is closely related to the partially sequenced and poorly characterized ilarvirus BLTaIV, which was found in an NGS dataset from blacklegged tick collected in the USA East Coast [15] (see CP phylogenetic tree, Figure 3).

**Figure 4:**
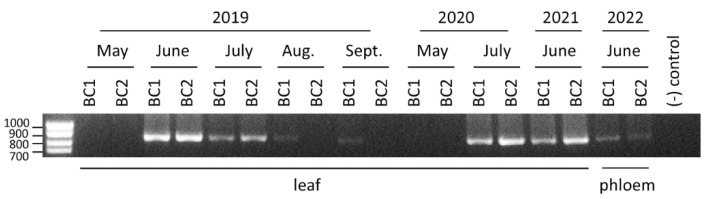
Detection of AIV2 by RT-PCR. Leaf or phloem tissue samples were collected from two apple trees (BC1 and BC2) from 2019 to 2022. AIV2 RNA3 was detected using RT-PCR and primers AIV2-3-F1223 and AIV2-3-R2073 (see Table S2 for primers sequence). The expected size of the PCR product is 851 base pairs. Migration of molecular mass markers is shown on the left.

So far, all well-characterized ilarviruses have been reported from plants [16,18] and it is possible that the reported tick-associated ilarvirus was detected because of cross-contamination of the environmental tick samples with plant material, especially considering that it was only detected at low incidence in the tick NGS dataset [15]. Although unlikely due to its high prevalence in our samples, we also considered the possibility that detection of AIV2 could have been the result of cross-contamination of our leaf samples with ticks or similar arthropods (such as mites, which can be prevalent in high numbers in apple orchards under conducive environmental conditions). We collected leaf samples from two individual trees that tested positive in our initial tests (trees BC1 and BC2). Samples were collected at different times of the growing season for three consecutive years. AIV2 could be detected by RT-PCR from the two trees each year although it was most prevalent in June and July (Figure 4). AIV2 was also detected in phloem tissues (stems peeled from the outer and inner bark, see Material and Methods) in a fourth consecutive year (Figure 4). These phloem-enriched samples are unlikely to be contaminated with arthropods. We conclude that AIV2 is most likely a plant virus. 

## 3. Discussion

Similar to previous reports from apple growing areas affected by rapid decline in NY, PA, and WA states in the USA [1,4,10], we detected multiple viruses in affected orchards in the Okanagan and Similkameen Valleys in BC. Irrespective of their status (RAD symptoms or asymptomatic), the majority of apple trees were infected by at least one virus or viroid, most often by two or more. Of the twelve viruses and one viroid tested by RT-PCR, up to eight could be detected in a single tree (Table 5). 

With the exception of AIV2, the most prevalent plant viruses/viroid detected by NGS in the BC samples were previously reported to infect apple trees. Analysis of the NGS contigs revealed that the BC isolates showed a high degree of sequence identity to reference isolates in some cases, but more variability in other cases. On one end of the spectrum, BC isolates of CCGaV were highly similar to the reference CCGaV isolate from citrus trees (99–99.6% sequence identity, Table S1). Similarly, apple isolates of CCGaV reported from the neighboring WA state were also shown to be closely related to the citrus reference isolate, highlighting a low degree of diversity for this virus in spite of its ability to infect both citrus and apple trees [5]. On the other end of the spectrum, high variability was noted for BC isolates of ACLSV (82–95% nt sequence identity between the assembled contigs and the reference isolate) and ASPV (81–99% nt sequence identity), suggesting the simultaneous presence of multiple divergent strains in BC (Table S1). This is perhaps not surprising considering the ubiquitous presence of these viruses in apple orchards throughout the world and their long history of adaptation to various apple cultivars [9]. Finally, even though BC isolates of ALV1 showed a high degree of nucleotide sequence identity (91–99%) to the PA8 reference isolate, we noted the presence of an extended 3′ UTR, which was conserved in all BC isolates tested (Figure 1). Whether or not the divergent BC strains of some apple viruses differ in terms of their infectivity, virulence or transmissibility is not known. 

We also noted interesting similarities and differences in the relative prevalence of viruses in BC and in other apple growing regions of North America. Not surprisingly, the three well known latent viruses from the family *Betaflexiviridae* (ASPV, ACLSV, and ASGV) were found in high prevalence in BC, similar to previous observations in orchards from northeastern and northwestern USA [1,4,10]. Although these viruses have not been reported to cause significant disease in most apple cultivars [9], symptoms induced by these viruses alone or in combination with other viruses could vary with the specific virus strains. Another prevalent virus was CCGaV, which was first reported in apple, from the neighboring WA state [5]. As mentioned above, the BC isolates shared a high degree of sequence identity to previously reported WA isolates. It was, therefore, not surprising to also find this virus in high prevalence in BC. 

On the other hand, ApMV, which was reported in 64% of samples tested in WA [1], was only detected in 4% of the BC samples. It is possible that different apple cultivars have different susceptibility to ApMV, explaining at least in part the regional differences in virus detection. Indeed, the majority of our samples had Ambrosia scions (78 out of 148 samples), while the majority of the WA samples had Honey Crisp scions [1]. However, a report from NY state conducted on Honey Crisp scions also indicated a low incidence of ApMV [3]. Sampling methods could have also impacted the results. We only tested leaf samples, while both leaf and root samples were tested in WA. It should be noted, however, that ApMV was readily detected from the WA leaf samples and should have also been detectable from our leaf samples [1].

ALV1 was first described from PA orchards affected by rapid decline [4]. In PA, the virus was detected at high incidence from Fuji scions and was also detected from Gala and Gold Delicious scions, although at a lower incidence. We detected the virus in 18% of our samples. In neighboring WA, the virus was not detected by NGS in six samples tested although its overall incidence was not rigorously tested by RT-PCR [1]. While it is possible that the virus is generally less prevalent in the northwestern corner of North America (including BC) than in northeastern USA due to climate differences and the relative prevalence of a possible not-yet identified aphid vector, it should be noted that Fuji scions were not the predominant scion tested in the WA and BC studies [1] (Table 1). Thus, there may also be regional differences in ALV1 prevalence due to the predominance of certain apple cultivars over others.

The most prevalent virus detected in our BC samples was AIV2, an ilarvirus related to a partial sequence from environmental tick samples (approximately 90% nt sequence identity) [15], but clearly distinct from any known plant ilarviruses. AIV2 was not previously reported from plant samples, and was not reported in the recent apple tree NGS study from neighboring WA [1]. The origin of this virus is unclear. Although we cannot completely exclude the possibility that AIV2 may be an arthropod-infecting virus detected in our leaf samples because of cross-contamination with arthropods, we consider this unlikely given its high incidence in our leaf samples, the high depth of coverage in the NGS data and the detection of the virus in the same trees for four consecutive years, including in phloem-enriched samples, which are unlikely to be contaminated with arthropods. In contrast, blacklegged tick associated virus was only detected in a single sample in the environmental tick samples and the NGS contigs only allowed the assembly of a partial sequence [15]. It is, thus, likely that AIV2 is a plant-infecting virus. So far, all known ilarviruses infect plants, with many ilarviruses known to infect fruit trees [14,16]. The detection of a new apple-associated ilarvirus is, thus, not surprising. Although not tested in this study, it is possible that the virus is present in the environment, i.e., in the wild flora, and only recently infected apple trees. The next steps will be to investigate the incidence of AIV2 in other apple-growing regions in BC and elsewhere and to estimate its host range and specific impact on apple tree health status through the construction of infectious clones.

Similar to other studies [1,4,10], we could not show a clear association between the presence of a single virus (or combination of viruses) and the tree health status (Table 6). However, it should be noted that infection with viruses can precede the appearance of symptoms. In addition, the intensity of virus-induced symptoms or their ability to be transmitted can be influenced by environmental conditions, such as temperature and water availability [19,20,21,22]. Finally, possible synergistic interactions among viruses can also greatly impact the outcome of infection [23,24,25]. The high prevalence of mixed infections in apple trees noted in this study (Table 5) and in previous studies [1,4,10] could facilitate such synergistic interactions and contribute to disease severity. Construction of infectious clones of representative BC isolates of the most prevalent viruses, including AIV2, will be necessary to assess their virulence in apple trees in single infection or in mixed infection with other viruses and in combination with various abiotic stresses and environmental conditions.

## 4. Materials and Methods

### 4.1. Sample Collection and Preparation

During the growing seasons of 2019 and 2020, 148 leaf samples were collected from apple trees from nine orchards in Summerland in the southern Okanagan Valley and from one orchard in Cawston in the Similkameen Valley (Table 1). Each sample combined ten leaves collected from three to four different branches of a single tree. More than half of the samples were collected from the scion/rootstock combination Ambrosia/M9, as it is the most planted variety in this area and many of these trees displayed severe RAD symptoms. Most other samples were collected from other major varieties, such as Ambrosia/Nic29, Honey Crisp/M9, Salish/M9, and Gala/M9. We also collected some samples from 14 minor scion varieties (Spartan, Fuji, Pink Lady, G. Reinette, McIntosh, Rein Russ, Splendour, Bisbee RB, Winter Banana, and the novel proprietary crosses 8S-54-28, 8S-06-28, 8S-54-21, 8S-56-50 and 9C-06-140), and 17 minor rootstocks (PiAu 51-11, PiAu 9-90, Bud 9, Bud 10, Bud 64-194, Bud 67-5-32, Bud 70-6-8, Bud 70-20-21, Bud 71-7-22, G11, G935N, G4124, G41N, G2034, G202 TC, M26 EMLA, and Supp3). Eighty leaf samples were collected from trees that had typical symptoms of RAD including cankers, peeling bark and/or necrosis around and above the graft union, leaf dwarfing and/or yellowing. The other 68 leaf samples were collected from non-symptomatic trees. Stem samples were also collected to test the presence of AIV2 in phloem tissues. Stems were first stripped from the outer layer of bark, and then peeled from the innermost bark layer to obtain samples that mostly consisted of phloem tissues.

Samples were ground into fine powder with mortar and pestle in liquid nitrogen and stored in conical tubes in a −80 °C freezer. Total nucleic acids were extracted from the stored leaf samples or phloem tissues using a modified protocol based on the SPECTRUM Plant Total RNA kit (Sigma-Aldrich, St. Louis, MO, USA) [26]. The quality and concentration of the RNA preparations were assessed with a NanoDrop spectrophotometer (ND-1000, Thermo Fisher Scientific, Waltham, MA, USA) at wavelengths of 230, 260, and 280 nm. All nucleic acid samples isolated had A260/280 and A260/230 ratio of 2.0 or above. The quality of nuclei acid samples used for next-generation sequencing (NGS) were further evaluated using the BioAnalyzer system (Agilent technologies, Santa Clara, CA, USA).

### 4.2. High Throughput Sequencing and Analyses

To maximize the chances of detecting all viruses present in the sampled orchards, ten composite samples were analyzed by NGS (Table 2). Each NGS samples combined the total RNAs from three individual leaf samples, thus representing a total of 30 individual leaf samples. The 30 individual samples were selected so that the main scion and rootstock varieties, sampling times (spring and summer), and geographical locations were included. We also tested samples collected from both trees that displayed typical RAD symptoms and trees that did not. Total RNAs were sent to Novogene (Sacramento, CA, USA). A cDNA library was prepared after ribosomal RNA depletion according to standard protocols. Sequencing was carried out by Novogene on an Illumina NovaSeq 6000 sequencer generating 150-bp paired-end reads.

The NGS data were analyzed using the CLC Genomics Workbench 20.0.4 (Qiagen, Hilden, Germany). The raw sequencing reads were filtered to remove adaptors sequences and reads of low quality, and then mapped to the reference genome of *Malus domestica* (GDDH13v1.1) to eliminate host sequences. The unmapped reads were *de novo* assembled into contigs, which were then blasted against a database consisting of reference sequences for all known plant viruses and viroids. Since the database used was downloaded into CLC, it was not regularly updated. Therefore, the sequences of contigs that had between 70–85% sequence identities to the closest hit of known viruses were separately blasted against the NCBI database to ensure that newly reported viruses were not missed. The number of reads for each detected virus was calculated by mapping the reads to the reference virus sequences.

### 4.3. Analysis of Virus Prevalence Using RT-PCR

Based on the NGS results, the 13 most prevalent viruses and viroid were selected for RT-PCR testing of individual leaf samples. Previously published primers were used for ASGV, ALV1 and ArWaV2 (Table S2) [4,27]. Specific primers were designed for each other virus/viroid based on the consensus region of all complete genome sequences available on the NCBI database (Table S2). In some cases, degenerate primers were designed to enable the detection of divergent virus isolates in a single PCR reaction. For the newly reported AIV2, primers were designed based on the assembled sequences from the NGS data and on the partial sequence of the closely related blacklegged tick-associated ilarvirus. First step reverse transcription was done using the SuperScript VILO Master Mix (Thermo Fisher Scientific) as described by the supplier. PCR was conducted using the recombinant Taq DNA Polymerase (Thermo Fisher Scientific) as described by the supplier except for using 40 reaction cycles. The PCR products were analyzed on 1.5% agarose gel, followed by staining with ethidium bromide.

### 4.4. Determination of the Complete Genome Sequence of AIV2 

To determine the 5′-terminal nucleotide of the three AIV2 RNAs, 5′ RACE was conducted using both poly(C)- and poly(G)-tailing. Briefly, two reverse primers and one forward primer were designed for each of the three genomic RNAs based on partial sequences obtained from NGS (Table S4). cDNAs were prepared using specific primers and the SuperScript VILO Master Mix. Poly(C)- and poly(G)-tailing of the resulting cDNAs were completed using terminal deoxynucleotidyl transferase (New England BioLabs, Ipswich, MA, USA). This was followed by PCR amplification with Q5 High-Fidelity DNA Polymerase (New England BioLabs) using the first specific reverse primer for each of the three RNAs and forward primer dG-14 or dC-13 (Table S4) for the first-round PCR. The second specific reverse primers for each of the three RNAs and forward primer WX29 [28] were used for the nested PCR. The resulting RT-PCR products were analyzed through electrophoresis on 1% agarose gel and amplicons of the expected sizes were cloned into pGEM-T Easy Vector (Promega, Madison, WI, USA). Positive clones were sequenced with M13 forward and reverse primers.

For determination of the RNAs 3′-terminal nucleotide, 3′ RACE was conducted using poly(U)-tailing and primers from the FirstChoice® RLM-RACE Kit (Thermo Fisher Scientific). Briefly, two forward primers and one reverse primer were designed for each RNA based on partial sequences obtained from NGS (Table S4). Poly(U)-tailing was first performed on total RNAs using the poly(A) polymerase (Thermo Fisher Scientific). cDNAs were then prepared using an adapted 3′ RACE A-Adapter (Table S4) and the SuperScript VILO Master Mix. This was followed by PCR amplification using Q5 High-Fidelity DNA Polymerase and the first specific forward primers for each of the three RNAs and the 3′ RACE outer primer (Table S4) for the first-round PCR. The second specific forward primers for each of the three RNAs and the 3′ RACE inner primer (Table S4) were used for the nested PCR. RT-PCR products of the expected size were cloned and sequenced as above.

To confirm the draft genome sequences of AIV2, forward and reverse primers were designed based on the 5′ and 3′ RACE results for each of three RNAs and used to produce cDNA fragments corresponding to the full length of each RNA using the SuperScript VILO Master Mix (Table S4). PCR was then conducted using Q5 High-Fidelity DNA Polymerase as described by the supplier. The resulting PCR products were cloned into pGEM-T Easy Vector and sequenced using M13 forward and reverse primers and virus specific primers.

### 4.5. Phylogenetic Analyses

Phylogenetic relationships were analyzed using MEGA X [29]. Amino acid sequences were aligned using Clustal W as implemented in MEGA X. Phylogenetic trees were then generated using the maximum likelihood method and default parameters. Bootstrap analyses (1000 replicates) were conducted to test the validity of the branches.

## Figures and Tables

**Table 1:** Information on the 148 individual leaf samples.

Orchards *	Number of Samples	Scions	Number of Samples	Rootstocks	Number of Samples	RAD Symptoms	Number of Samples
A	14	Ambrosia	78	M9	104	Yes	80
B	14	Honey Crisp	26	Nic29	19	No	68
C	30	Salish	10	Others (17)	25		
D	19	Gala	12				
E	13	Others (14)	19				
F	15	Unknown	3				
G	10						
H	10						
I	20						
J	3						

* Most orchards were in Summerland (Okanagan valley) except for orchard I, which was in Cawston (Similkameen valley).

**Table 2:** Information on the ten composite leaf samples analyzed by NGS.

NGS Samples *	Orchards	Scions	Rootstocks	RAD Symptoms **
HX1	B	Fuji, Honey Crispy, 8S-54-28	M9	Yes (3)
HX2	A	Ambrosia, Spartan	M9	Yes (3)
HX3	D, E, F	Ambrosia, Honey Crisp	M9, Nic29	Yes (2), No (1)
HX4	I, J	Ambrosia, Pink Lady	M9, G11	Yes (3)
HX5	C	Honey Crisp	G935N, Bud9, Bud 71-7-22	No (2), Yes (1)
HX6	C	Honey Crisp	G4124, G2034, Bud9	No (3)
HX7	C	G. Reinette, Mcintosh, Rein Russ	M9	No (2), Yes (1)
HX8	C	Winter banana, Splendour, Bisbee RD	M9	No (3)
HX9	B	8S-06-28, 8S-56-50, 9C-06-140	M9	Yes (3)
HX10	A, E	Ambrosia	M9	Yes (2), No (1)

* Each composite NGS sample was a combination of three leaf samples taken from three trees. ** Number of leaf samples collected from trees displaying RAD symptoms (Yes) or not (No).

**Table 3:** Plant viruses detected by NGS.

Acronym	Virus or Viroid Name	Species Name	GenBank Accession Number of Reference Isolate	Genus	Family
AHVd	Apple hammerhead viroid	*Apple hammerhead viroid*	NC_028132.1	*Pelamoviroid*	*Avsunviroidae*
ACLSV	Apple chlorotic leaf spot virus	*Apple chlorotic leaf spot virus*	NC_001409.1	*Trichovirus*	*Betaflexiviridae*
ASGV	Apple stem grooving virus	*Apple stem grooving virus*	NC_001749.2	*Capillovirus*	
CVA	Cherry virus A	*Cherry virus A*	NC_003689.1		
ASPV	Apple stem pitting virus	*Apple stem pitting virus*	NC_003462.2	*Foveavirus*	
ALV1	Apple luteovirus 1	*Apple luteovirus 1*	NC_040680.1	*Luteovirus*	*Tombusviridae*
TCV	Turnip crinkle virus	*Turnip crinkle virus*	NC_003821.3	*Betacarmovirus*	
ARWaV-1	Apple rubbery wood virus 1	*Apple rubodvirus 1*	NC_055390.1, NC_055391.1, NC_055392.1	*Rubodvirus*	*Phenuiviridae*
ARWaV-2	Apple rubbery wood virus 2	*Apple rubodvirus 2*	NC_055533.1, NC_055534.1, NC_055535.1, NC_055536.1, NC_055537.1		
CCGaV	Citrus concave gum-associated virus	*Citrus coguvirus*	NC_035759.1, NC_035454.1	*Coguvirus*	
AIV2	Apple ilarvirus 2	Identified in this study, not classified	ON932434, ON932435, ON932436	*Ilarvirus*	*Bromoviridae*
ApMV	Apple mosaic virus	*Apple mosaic virus*	NC_003464.1, NC_003465.1, NC_003480.1		
SNIV	Solanum nigrum ilarvirus	Not classified	MN216370.1, MN216373.1, MN216376.1		
TSV	Tobacco streak virus	*Tobacco streak virus*	NC_003844.1, NC_003842.1, NC_003845.1		
AIV1	Apple ilarvirus 1	Not classified	MN386957.1, MN386958.1		
PNRSV	Prunus necrotic ringspot virus	*Prunus necrotic ringspot virus*	NC_004362.1, NC_004363.1, NC_004364.1		
AlMV	Alfalfa mosaic virus	*Alfalfa mosaic virus*	NC_001495.1, NC_002024.2, NC_002025.1	*Alfamovirus*	
CYMV	Clover yellow mosaic virus	*Clover yellow mosaic virus*	NC_001753.1	*Potexvirus*	*Alphaflexiviridae*
WClMV	White clover mosaic virus	*White clover mosaic virus*	NC_003820.1		
TVCV	Turnip vein clearing virus	*Turnip vein-clearing virus*	NC_001873.1	*Tobamovirus*	*Virgaviridae*
TuMV	Turnip mosaic virus	*Turnip mosaic virus*	NC_002509.2	*Potyvirus*	*Potyviridae*
SoMV	Sowbane mosaic virus	*Sowbane mosaic virus*	NC_011187.1	*Sobemovirus*	*Solemoviridae*

**Table 4:** Viruses detected by NGS in each composite sample.

NGS Samples	HX1	HX2	HX3	HX4	HX5	HX6	HX7	HX8	HX9	HX10
Total reads	103,066,652	81,797,780	101,947,128	86,362,078	81,880,242	102,784,288	71,349,254	94,601,674	85,908,874	77,797,014
Reads Mapped to *Malus Domestica*	97,549,197	75,364,116	95,651,348	80,689,725	72,958,929	92,960,006	65,850,694	87,214,426	79,122,763	71,389,255
Reads not Mapped to *M. domestica*	5,517,455	6,433,664	6,295,780	5,672,353	8,921,313	9,824,282	5,498,560	7,387,248	6,786,111	6,407,759
Reads Mapped to Plant Viruses	40,286	161	132,732	52,869	1994	986	148,093	357,639	4838	76,969
AHVd			8699	3435	704	54	63,949	180,962		200
ACLSV			12,174	20,423	8		12,801	10,955		677
ASGV			9785	4467						2643
CVA	187									
ASPV		5	35,542	11,105	8	12	43,759	151,192		6532
ALV1	39,292		12,494	1978			10,409			7413
ARWaV-1				3840			15,168		3970	
ARWaV-2			41,807	6141			232			874
CCGaV			10,782			9		12,967		56,775
AIV2 *	114	156	1389	120	1257	882	1656	1521	848	1652
ApMV				931						
SNIV	110			211			30	36	14	22
TSV				202						
AIV1			81							10
PNRSV	250									
AlMV	16		14		10	14				
CYMV	149			10	7	7	71			
WClMV	116					8	18	6		
TVCV	40			6						149
TuMV	12		46						6	
TCV										10
SoMV										22

* Reads were mapped to the complete sequenced genome of the virus, as determined later by Sanger sequencing. See Table 3 for definition of virus acronyms.

**Table 5:** Number of viruses and/or viroid simultaneously detected in individual samples.

Number of Viruses and/or Viroid	Number of Samples	Percentage of Samples (%)
0	1	0.7
1	26	17.6
2	26	17.6
3	24	16.2
4	15	10.1
5	29	19.6
6	15	10.1
7	9	6.1
8	3	2.0

**Table 6:** Prevalence of selected viruses in apple orchards of the Okanagan and Similkameen valleys.

Viruses Tested	RAD Symptoms (80) *		No RAD Symptoms (68)		All Samples (148)	
	No. of Positive Samples	Percentage of Positive Samples (%)	No. of Positive Samples	Percentage of Positive Samples (%)	No. of Positive Samples	Percentage of Positive Samples (%)
AIV2	46	57.5	48	70.6	94	63.5
CCGaV	49	61.3	43	63.2	92	62.2
ASPV	43	53.8	38	55.9	81	54.7
ACLSV	36	45.0	38	55.9	74	50.0
ASGV	34	42.5	28	41.2	62	41.9
AHVd	15	18.8	19	27.9	34	23.0
ALV1	15	18.8	12	17.6	27	18.2
ARWaV2	14	17.5	9	13.2	23	15.5
TSV	6	7.5	8	11.8	14	9.5
ARWaV1	8	10.0	5	7.4	13	8.8
SNIV	7	8.8	4	5.9	11	7.4
ApMV	5	6.3	1	1.5	6	4.1
PNRSV	1	1.3	0	0.0	1	0.7

* Numbers in parentheses indicate the number of samples tested.

## Data Availability

Nucleotide sequence of near full-length genome contigs of ALV1 BC isolates have been deposited under NCBI numbers OP271661, OP271662 and OP271663. The full-length sequence of the three AIV2RNAs have been deposited under NCBI numbers ON932434 to ON932436. These sequences are also available as Text S1–S4.

## Supplementary Materials

The following supporting information can be downloaded at: www.mdpi.com/article/10.3390/pathogens11111231/s1, Figure S1: Multiple sequence alignment of the deduced amino acid sequences of ORF5, ORF5a and ORF8 from PA8 and BC isolates of ALV1; Figure S2: Original uncropped gel pictures for Figure 1B and Figure 4; Table S1: Sequence reads, percentage of nucleotide identity and percentage of genome coverage for viruses detected with NGS; Table S2: Primers used for RT-PCR detection of the most prevalent viruses; Table S3: Ilarvirus names, acronyms and accession numbers; Table S4: Primers used for the cloning and sequencing of AIV2. Texts S1–S3: Annotated genome sequence of ALV1 isolates BC52, BC 85 and BC134, respectively. Text S4: Annotated genome sequence of AIV2.
