# Supplementary material for: Characterizing the Virome of Apple Orchards Affected by Rapid Decline in the Okanagan and Similkameen Valleys of British Columbia (Canada)
Source: Pathogens. 2022 Oct 25;11(11):1231. doi: 10.3390/pathogens11111231 (PMC9698585; doi:10.3390/pathogens11111231)
Supplement: Supplementary file 1 [file pathogens-11-01231-s001.zip › Figure S1.pptx]

## Slide 1
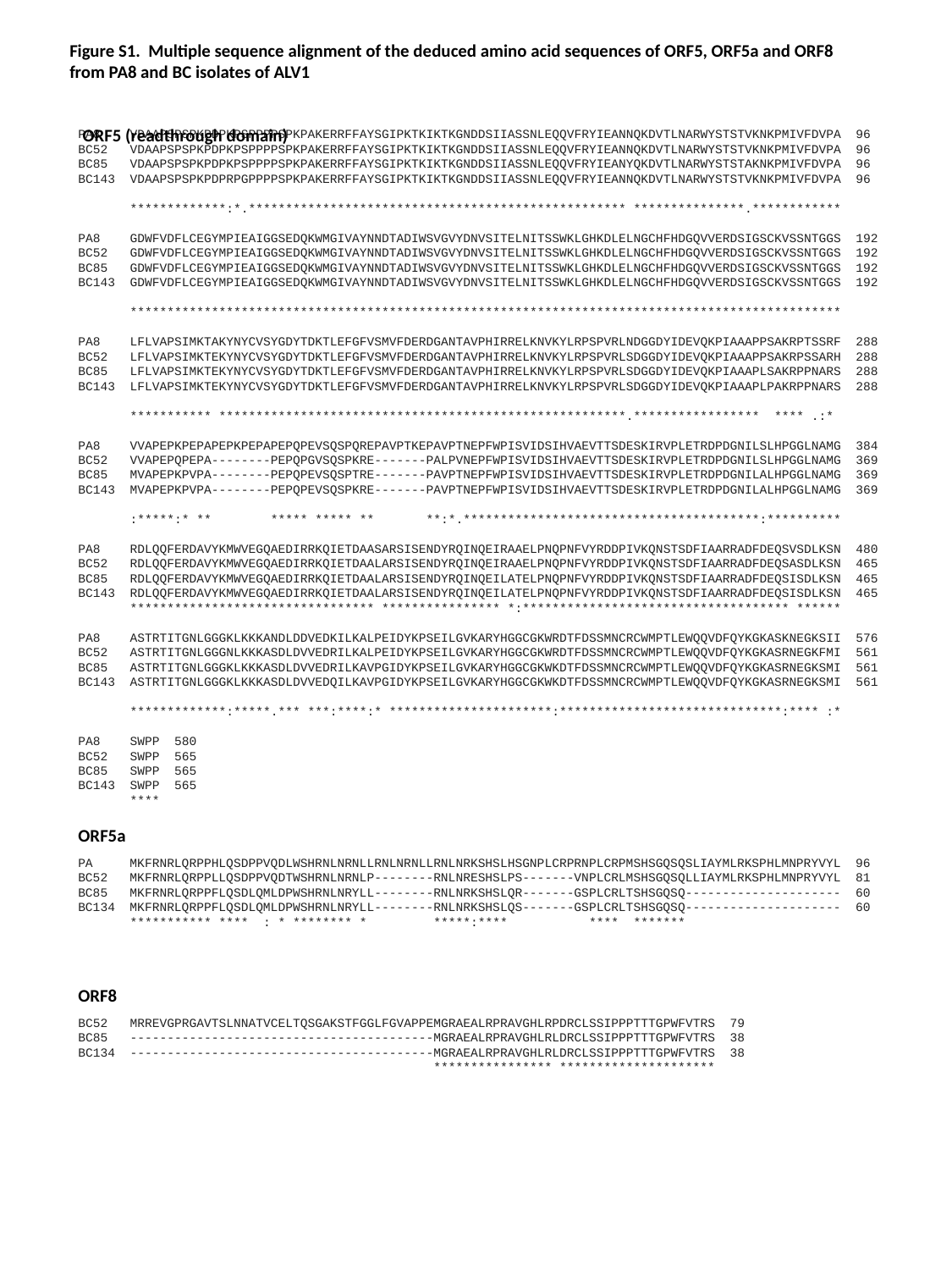

Figure S1. Multiple sequence alignment of the deduced amino acid sequences of ORF5, ORF5a and ORF8 from PA8 and BC isolates of ALV1
ORF5 (readthrough domain)
PA8 VDAAPSPSPKPDPKPSPPPPSPKPAKERRFFAYSGIPKTKIKTKGNDDSIIASSNLEQQVFRYIEANNQKDVTLNARWYSTSTVKNKPMIVFDVPA 96
BC52 VDAAPSPSPKPDPKPSPPPPSPKPAKERRFFAYSGIPKTKIKTKGNDDSIIASSNLEQQVFRYIEANNQKDVTLNARWYSTSTVKNKPMIVFDVPA 96
BC85 VDAAPSPSPKPDPKPSPPPPSPKPAKERRFFAYSGIPKTKIKTKGNDDSIIASSNLEQQVFRYIEANYQKDVTLNARWYSTSTAKNKPMIVFDVPA 96
BC143 VDAAPSPSPKPDPRPGPPPPSPKPAKERRFFAYSGIPKTKIKTKGNDDSIIASSNLEQQVFRYIEANNQKDVTLNARWYSTSTVKNKPMIVFDVPA 96
 *************:*.*************************************************** ***************.************
PA8 GDWFVDFLCEGYMPIEAIGGSEDQKWMGIVAYNNDTADIWSVGVYDNVSITELNITSSWKLGHKDLELNGCHFHDGQVVERDSIGSCKVSSNTGGS 192
BC52 GDWFVDFLCEGYMPIEAIGGSEDQKWMGIVAYNNDTADIWSVGVYDNVSITELNITSSWKLGHKDLELNGCHFHDGQVVERDSIGSCKVSSNTGGS 192
BC85 GDWFVDFLCEGYMPIEAIGGSEDQKWMGIVAYNNDTADIWSVGVYDNVSITELNITSSWKLGHKDLELNGCHFHDGQVVERDSIGSCKVSSNTGGS 192
BC143 GDWFVDFLCEGYMPIEAIGGSEDQKWMGIVAYNNDTADIWSVGVYDNVSITELNITSSWKLGHKDLELNGCHFHDGQVVERDSIGSCKVSSNTGGS 192
 ************************************************************************************************
PA8 LFLVAPSIMKTAKYNYCVSYGDYTDKTLEFGFVSMVFDERDGANTAVPHIRRELKNVKYLRPSPVRLNDGGDYIDEVQKPIAAAPPSAKRPTSSRF 288 BC52 LFLVAPSIMKTEKYNYCVSYGDYTDKTLEFGFVSMVFDERDGANTAVPHIRRELKNVKYLRPSPVRLSDGGDYIDEVQKPIAAAPPSAKRPSSARH 288
BC85 LFLVAPSIMKTEKYNYCVSYGDYTDKTLEFGFVSMVFDERDGANTAVPHIRRELKNVKYLRPSPVRLSDGGDYIDEVQKPIAAAPLSAKRPPNARS 288 BC143 LFLVAPSIMKTEKYNYCVSYGDYTDKTLEFGFVSMVFDERDGANTAVPHIRRELKNVKYLRPSPVRLSDGGDYIDEVQKPIAAAPLPAKRPPNARS 288
 *********** *******************************************************.***************** **** .:*
PA8 VVAPEPKPEPAPEPKPEPAPEPQPEVSQSPQREPAVPTKEPAVPTNEPFWPISVIDSIHVAEVTTSDESKIRVPLETRDPDGNILSLHPGGLNAMG 384
BC52 VVAPEPQPEPA--------PEPQPGVSQSPKRE-------PALPVNEPFWPISVIDSIHVAEVTTSDESKIRVPLETRDPDGNILSLHPGGLNAMG 369
BC85 MVAPEPKPVPA--------PEPQPEVSQSPTRE-------PAVPTNEPFWPISVIDSIHVAEVTTSDESKIRVPLETRDPDGNILALHPGGLNAMG 369
BC143 MVAPEPKPVPA--------PEPQPEVSQSPKRE-------PAVPTNEPFWPISVIDSIHVAEVTTSDESKIRVPLETRDPDGNILALHPGGLNAMG 369
 :*****:* ** ***** ***** ** **:*.****************************************:**********
PA8 RDLQQFERDAVYKMWVEGQAEDIRRKQIETDAASARSISENDYRQINQEIRAAELPNQPNFVYRDDPIVKQNSTSDFIAARRADFDEQSVSDLKSN 480
BC52 RDLQQFERDAVYKMWVEGQAEDIRRKQIETDAALARSISENDYRQINQEIRAAELPNQPNFVYRDDPIVKQNSTSDFIAARRADFDEQSASDLKSN 465
BC85 RDLQQFERDAVYKMWVEGQAEDIRRKQIETDAALARSISENDYRQINQEILATELPNQPNFVYRDDPIVKQNSTSDFIAARRADFDEQSISDLKSN 465
BC143 RDLQQFERDAVYKMWVEGQAEDIRRKQIETDAALARSISENDYRQINQEILATELPNQPNFVYRDDPIVKQNSTSDFIAARRADFDEQSISDLKSN 465
 ********************************* **************** *:************************************ ******
PA8 ASTRTITGNLGGGKLKKKANDLDDVEDKILKALPEIDYKPSEILGVKARYHGGCGKWRDTFDSSMNCRCWMPTLEWQQVDFQYKGKASKNEGKSII 576
BC52 ASTRTITGNLGGGNLKKKASDLDVVEDRILKALPEIDYKPSEILGVKARYHGGCGKWRDTFDSSMNCRCWMPTLEWQQVDFQYKGKASRNEGKFMI 561
BC85 ASTRTITGNLGGGKLKKKASDLDVVEDRILKAVPGIDYKPSEILGVKARYHGGCGKWKDTFDSSMNCRCWMPTLEWQQVDFQYKGKASRNEGKSMI 561
BC143 ASTRTITGNLGGGKLKKKASDLDVVEDQILKAVPGIDYKPSEILGVKARYHGGCGKWKDTFDSSMNCRCWMPTLEWQQVDFQYKGKASRNEGKSMI 561
 *************:*****.*** ***:****:* **********************:******************************:**** :*
PA8 SWPP 580
BC52 SWPP 565
BC85 SWPP 565
BC143 SWPP 565
 ****
ORF5a
PA MKFRNRLQRPPHLQSDPPVQDLWSHRNLNRNLLRNLNRNLLRNLNRKSHSLHSGNPLCRPRNPLCRPMSHSGQSQSLIAYMLRKSPHLMNPRYVYL 96
BC52 MKFRNRLQRPPLLQSDPPVQDTWSHRNLNRNLP--------RNLNRESHSLPS-------VNPLCRLMSHSGQSQLLIAYMLRKSPHLMNPRYVYL 81
BC85 MKFRNRLQRPPFLQSDLQMLDPWSHRNLNRYLL--------RNLNRKSHSLQR-------GSPLCRLTSHSGQSQ--------------------- 60
BC134 MKFRNRLQRPPFLQSDLQMLDPWSHRNLNRYLL--------RNLNRKSHSLQS-------GSPLCRLTSHSGQSQ--------------------- 60
 *********** **** : * ******** * *****:**** **** *******
ORF8
BC52 MRREVGPRGAVTSLNNATVCELTQSGAKSTFGGLFGVAPPEMGRAEALRPRAVGHLRPDRCLSSIPPPTTTGPWFVTRS 79
BC85 -----------------------------------------MGRAEALRPRAVGHLRLDRCLSSIPPPTTTGPWFVTRS 38
BC134 -----------------------------------------MGRAEALRPRAVGHLRLDRCLSSIPPPTTTGPWFVTRS 38
 **************** *********************
