# Supplementary material for: Characterizing the Virome of Apple Orchards Affected by Rapid Decline in the Okanagan and Similkameen Valleys of British Columbia (Canada)
Source: Pathogens. 2022 Oct 25;11(11):1231. doi: 10.3390/pathogens11111231 (PMC9698585; doi:10.3390/pathogens11111231)
Supplement: Supplementary file 1 [file pathogens-11-01231-s001.zip › Figure S2.pptx]

## Slide 1
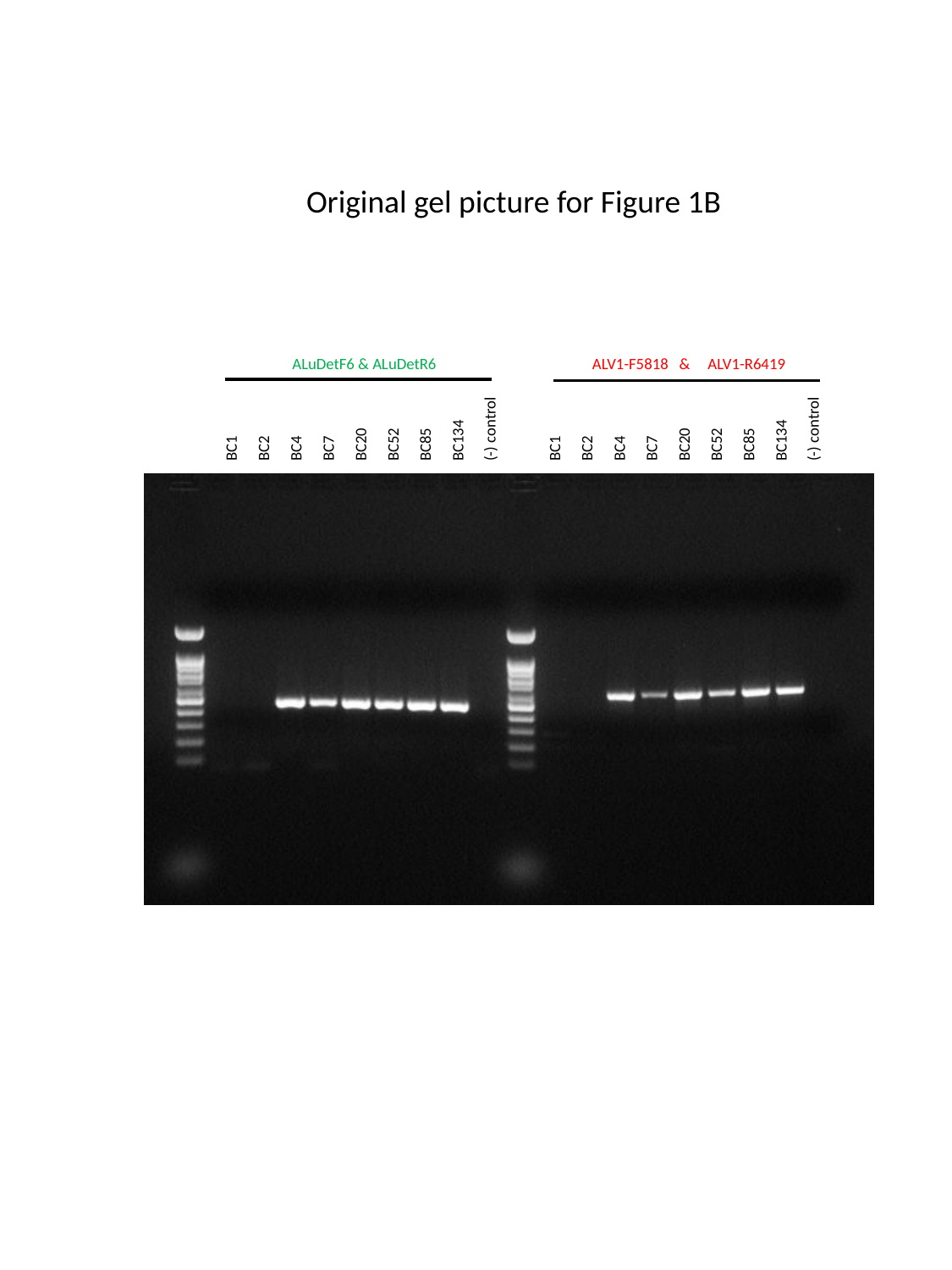

BC1
BC2
BC4
BC7
BC20
BC52
BC85
BC134
(-) control
BC1
BC2
BC4
BC7
BC20
BC52
BC85
BC134
(-) control
Original gel picture for Figure 1B
ALuDetF6 & ALuDetR6
ALV1-F5818 & ALV1-R6419

## Slide 2
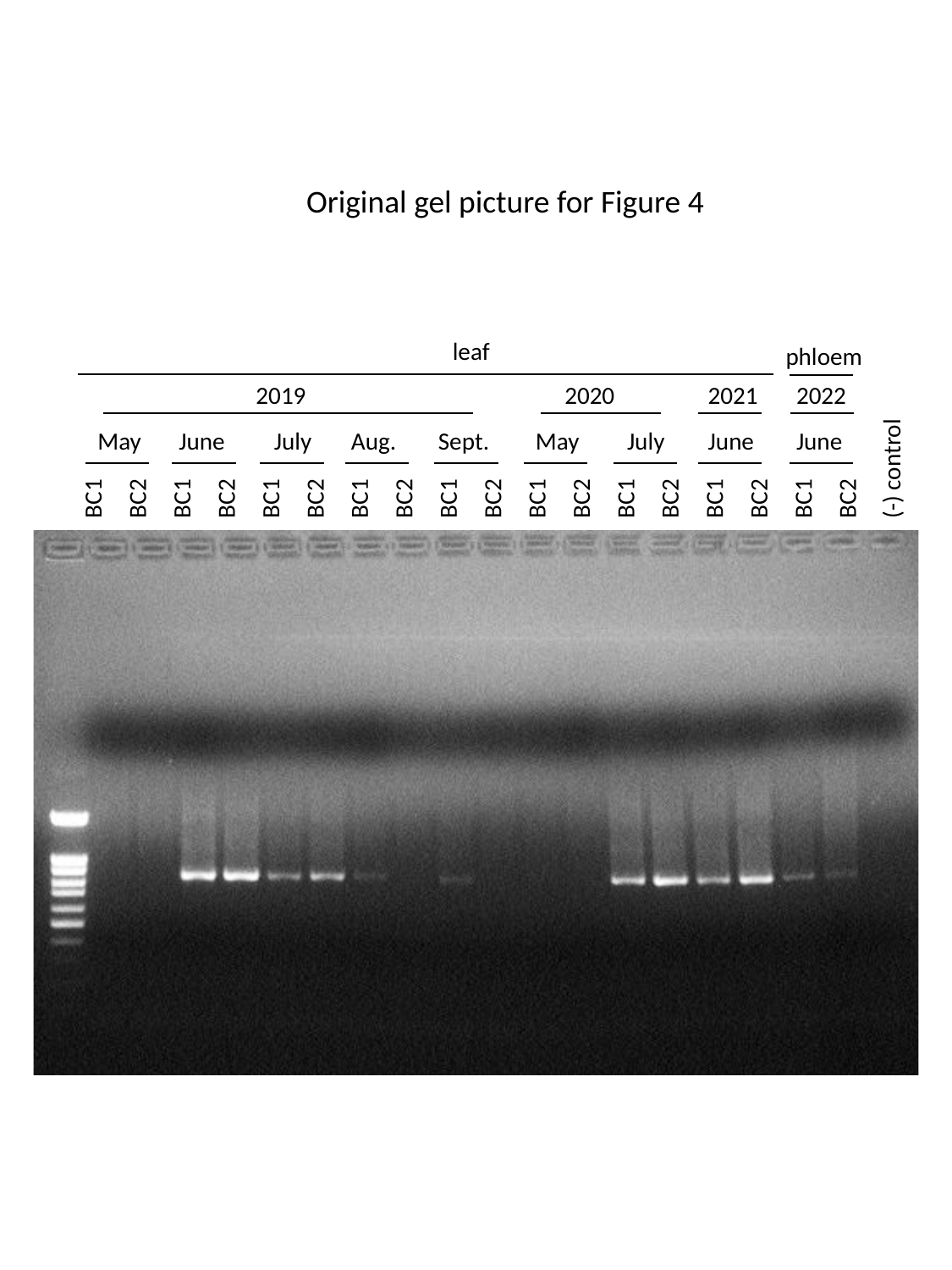

BC1
BC2
BC1
BC2
BC1
BC2
BC1
BC2
BC1
BC2
BC1
BC2
BC1
BC2
BC1
BC2
BC1
BC2
(-) control
Original gel picture for Figure 4
leaf
phloem
2019
2020
2021
2022
May
June
July
Aug.
Sept.
May
July
June
June
