# Supplementary material for: Characterizing the Virome of Apple Orchards Affected by Rapid Decline in the Okanagan and Similkameen Valleys of British Columbia (Canada)
Source: Pathogens. 2022 Oct 25;11(11):1231. doi: 10.3390/pathogens11111231 (PMC9698585; doi:10.3390/pathogens11111231)
Supplement: Supplementary file 1 [file pathogens-11-01231-s001.zip › Text S1.pdf]

```
Text S1. Annotated sequence of ALV1 isolate BC52 as submitted to NCBI

LOCUS       Seq1                    6362 bp    RNA     linear   VRL 19-AUG-
2022
DEFINITION  Apple luteovirus 1 isolate BC52, near complete genome.
ACCESSION   Seq1
VERSION
KEYWORDS    .
SOURCE      Apple luteovirus 1
  ORGANISM  Apple luteovirus 1
            Viruses; Riboviria; Orthornavirae; Kitrinoviricota;
            Tolucaviricetes; Tolivirales; Tombusviridae; Luteovirus.
REFERENCE   1  (bases 1 to 6362)
  AUTHORS   Xiao,H., Hao,W., Storoschuk,G., MacDonald,J. and Sanfacon,H.
  TITLE     Virus prevalence in apple orchards affected by rapid decline and
            identification of a novel highly prevalent ilarvirus in the
            Okanagan valley of British Columbia (Canada)
  JOURNAL   unpublished
REFERENCE   2  (bases 1 to 6362)
  AUTHORS   Xiao,H. and Sanfacon,H.
  TITLE     Direct Submission
  JOURNAL   Submitted (19-AUG-2022) Summerland Research and Development
Centre,
            Agriculture & Agri-Food Canada, 4200 Highway 97, Summerland
            Research and Development Centre, SUMMERLAND, BC V0H 1Z0, Canada
COMMENT     Bankit Comment: ALT EMAIL:huogen@gmail.com
```

```
            Bankit Comment: TOTAL # OF SEQS:1

            ##Assembly-Data-START##
            Assembly Method       :: CLC Genomic Work Bench 20 v. 20.0.4
            Sequencing Technology :: Illumina
            ##Assembly-Data-END##
FEATURES             Location/Qualifiers
     source          1..6362
                     /organism="Apple luteovirus 1"
                     /mol_type="genomic RNA"
                     /isolate="BC52"
                     /host="Malus domestica"
                     /db_xref="taxon:2170544"
                     /country="Canada"
                     /collection_date="28-August-2019"
     gene            90..2950
                     /gene="ORF1-ORF2"
     CDS             join(90..1361,1361..2950)
                     /gene="ORF1-ORF2"
                     /ribosomal_slippage
                     /note="RNA dependent RNA polymerase P1-P2 fusion; -1
                     ribosomal frameshift slippage"
                     /codon_start=1
                     /product="ORF1-ORF2"
/translation="MLFDDLICASFKVVKDFISHIYNNLRTVYKKFKVWLWELQGKFS

QHDAFVDACYGYMDDVEQFEWDCYSAYNDADVELALARLHLDTVLKAPKVTGWPVPTR
```

```
PDGAPTTTEVPKYPTLHELAEKIRTSVRRERVFQAAGEASGDKDAEPESEVPEGWHVD

VWNRFQDEERTYWENYYANPIELAPQVIPVNPGVAEPPMPKPIYTERVAFTEDELFKA

EARLTRAKCSYSSTIEDIKDQYEEEKGEGYFGRFFNTFEQRMHYVKRARSRRAKTDQL

CHKVQGKLSQVAELPDFYELCTVREVETGEFHTVMDEGEEIKRPIVKVSRSIKPECRR

DAQSYIRKYIRSKNNRVGADEIGVATINRYVAQFADDMKLDMASSEFLARTALTIVPV

ITKQEMMQAMVIHSPAARKARADLAALEGQDFLEGLLTASGFESPFSILGLPEIVVRS

GCLPRKVKSRINYLSQFSLGLDYRIPNPSFHNALVAVERRVFTVGKGDDIVRPPKPRR

NIFEERLGYFRDKIVADVGPLRACSVAQLVSTYKSSKRRQYELAAFKLRKKPVCKEDA

DVTAFLKMEKHWMCKAIAPRLICPRSKRYNIELGRRLKLNEKRFMHAIDNVFGSATVL

SGYDNFKQGRLIAGKWNKFRNPVAIGVDASRFDQHVSTEALKWEHSIYNKVFGDPLLR

DLLDWQTVNKCSLFVEDKMLRFKVKGHRMSGDINTSMGNKLIMCGMMHNYFRELGVKA

ELCNNGDDCVIICERKDERKFDGLGKWFWEYGFNMAIEPPVYSLAKLEFCQSRPVCIN

GKYRMVRRPDSIAKDAHTMLSMQNAEDVKSFMSATGQCGMILNSGVPILDAYHSNLYR

GSGYKKVSESFIDRVISYGTDERLQGRRTRVEEPVTMENRLSYWDAFGVDPQTQVLVE
                     RYLNNLRIGCEPLGVKIVTPLLTSTLLEIPYYKPLNLAP"
     gene            90..1364
                     /gene="ORF1"
     CDS             90..1364
                     /gene="ORF1"
                     /note="RNA dependent RNA polymerase"
                     /codon_start=1
```

```
                     /product="P1"

/translation="MLFDDLICASFKVVKDFISHIYNNLRTVYKKFKVWLWELQGKFS

QHDAFVDACYGYMDDVEQFEWDCYSAYNDADVELALARLHLDTVLKAPKVTGWPVPTR

PDGAPTTTEVPKYPTLHELAEKIRTSVRRERVFQAAGEASGDKDAEPESEVPEGWHVD

VWNRFQDEERTYWENYYANPIELAPQVIPVNPGVAEPPMPKPIYTERVAFTEDELFKA

EARLTRAKCSYSSTIEDIKDQYEEEKGEGYFGRFFNTFEQRMHYVKRARSRRAKTDQL

CHKVQGKLSQVAELPDFYELCTVREVETGEFHTVMDEGEEIKRPIVKVSRSIKPECRR

DAQSYIRKYIRSKNNRVGADEIGVATINRYVAQFADDMKLDMASSEFLARTALTIVPV
                     ITKQEMMQAMVIHSPAARKARADLAALEGQDF"
     gene            226..1020
                     /gene="ORF0"
     CDS             226..1020
                     /gene="ORF0"
                     /codon_start=1
                     /product="putative P0 protein"

/translation="MTPSSMPVTVTWTTSSSSSGTATQHTMMLMLSLPSPDSTSTPSL

RLLKLPAGPSLQDLTVRQPRRRSPNTRLCTNSQRRSAQACDESGYFKLQVKRLVIKMQ

NLSLKCQRGGTLTSGTGSKTRSEPTGKITTPTPSSWRRRLYLSTQGSLNLPCPSRYTP

RESPLRRTSSSKPRLGSPGQNAHTRPPLKTSRTSMKKRRERATSAAFLTRLNSECIML
                     RERGAVEPRRTSCVTRFKVNSVRLLNYLISMSCVPSEKWKPVSSTL"
     gene            2947..3084
                     /gene="ORF3a"
```

```
     CDS             2947..3084
                     /gene="ORF3a"
                     /note="viral movement; alternative translation
initiation
                     at an AUA"
                     /codon_start=1
                     /transl_except="pos:2947..2949, aa:Met"
                     /product="P3a protein"

/translation="MDFHLLAGFFLGFLASIPITVCVCYVAYIKISQQVRSIVNEYGR
                     A"
     gene            3071..5371
                     /gene="ORF3-ORF5"
     CDS             3071..5371
                     /gene="ORF3-ORF5"
                     /note="read-through stop codon in ORF3"
                     /codon_start=1
                     /transl_except="pos:3671..3673, aa:Xaa"
                     /product="P3-P5 polyprotein"

/translation="MVVRRRQPIRRNIRRRRNGPRRFAAPPRVVVVPGRPRRRRRNGR

TNPRVNRGRITFSSRPAEVFTFTVDDLKAGSTGVLKFGPGLSQCAAVSGGVLKSYHQY

KIIGLTCGYVTNASSTTAGAFALEIDTTCSRSALESRIISFPVTKNTSKFFPPGVING

QNWISSDTNQFFLLYGGNGSKTEIAGQLLIKVMITLQGPK*VDAAPSPSPKPDPKPSP

PPPSPKPAKERRFFAYSGIPKTKIKTKGNDDSIIASSNLEQQVFRYIEANNQKDVTLN

ARWYSTSTVKNKPMIVFDVPAGDWFVDFLCEGYMPIEAIGGSEDQKWMGIVAYNNDTA
```

```
DIWSVGVYDNVSITELNITSSWKLGHKDLELNGCHFHDGQVVERDSIGSCKVSSNTGG

SLFLVAPSIMKTEKYNYCVSYGDYTDKTLEFGFVSMVFDERDGANTAVPHIRRELKNV

KYLRPSPVRLSDGGDYIDEVQKPIAAAPPSAKRPSSARHVVAPEPQPEPAPEPQPGVS

QSPKREPALPVNEPFWPISVIDSIHVAEVTTSDESKIRVPLETRDPDGNILSLHPGGL

NAMGRDLQQFERDAVYKMWVEGQAEDIRRKQIETDAALARSISENDYRQINQEIRAAE

LPNQPNFVYRDDPIVKQNSTSDFIAARRADFDEQSASDLKSNASTRTITGNLGGGNLK

KKASDLDVVEDRILKALPEIDYKPSEILGVKARYHGGCGKWRDTFDSSMNCRCWMPTL
                     EWQQVDFQYKGKASRNEGKFMISWPP*"
     gene            3071..3673
                     /gene="ORF3"
     CDS             3071..3673
                     /gene="ORF3"
                     /codon_start=1
                     /product="coat protein"

/translation="MVVRRRQPIRRNIRRRRNGPRRFAAPPRVVVVPGRPRRRRRNGR

TNPRVNRGRITFSSRPAEVFTFTVDDLKAGSTGVLKFGPGLSQCAAVSGGVLKSYHQY

KIIGLTCGYVTNASSTTAGAFALEIDTTCSRSALESRIISFPVTKNTSKFFPPGVING
                     QNWISSDTNQFFLLYGGNGSKTEIAGQLLIKVMITLQGPK"
     gene            3123..3647
                     /gene="ORF4"
     CDS             3123..3647
                     /gene="ORF4"
                     /codon_start=1
```

```
                     /product="movement protein"

/translation="MDLAGLQHRHGWLWSQDGLEDEEEMEEQTLELTEAELPSLRGQL

RSSLSQWTTSKPDPRGSSSSDRAYHSALRFQGEYSSPTINIKSSVSRAVMSRTPAAPL

PAHLLWRSTLPVLEAPLNQESFHSPSRRTLQNSSRRGSLMGRIGSALTRTNSSSSMEE
                     MDPRPRSRDSYSSR"
     gene            4473..4718
                     /gene="ORF5a"
     CDS             4473..4718
                     /gene="ORF5a"
                     /codon_start=1
                     /product="putative P5a protein"

/translation="MKFRNRLQRPPLLQSDPPVQDTWSHRNLNRNLPRNLNRESHSLP
                     SVNPLCRLMSHSGQSQLLIAYMLRKSPHLMNPRYVYL"
     gene            5359..5472
                     /gene="ORF6"
     CDS             5359..5472
                     /gene="ORF6"
                     /codon_start=1
                     /product="putative P6 protein"
                     /translation="MATVVYTPYLVAFIFKYCTYRSKMPKLSGVRPGRTGI"
     gene            5653..5802
                     /gene="ORF7"
     CDS             5653..5802
                     /gene="ORF7"
                     /codon_start=1
                     /product="putative P7 protein"
```

```
/translation="MENMGMMDLASPGVMKTAEMNARGCFRGSDALDVRSLLLSYTLP
                     VTAAR"
     gene            5824..6063
                     /gene="ORF8"
     CDS             5824..6063
                     /gene="ORF8"
                     /codon_start=1
                     /product="putative P8 protein"
/translation="MRREVGPRGAVTSLNNATVCELTQSGAKSTFGGLFGVAPPEMGR
                     AEALRPRAVGHLRPDRCLSSIPPPTTTGPWFVTRS"
BASE COUNT     1656 a   1543 c   1633 g   1530 t
ORIGIN
        1 cgatcatcac aaaccaaagc ccctcgcttt atctgtcagc ggtttagtct accgtctcag
       61 agtttaagtg tgaaaccaag cccgtcaaga tgttgtttga cgacctcatc tgcgctagtt
      121 tcaaggttgt aaaagatttc atctctcaca tctacaacaa cctacgcacc gtttacaaga
      181 aatttaaggt gtggctgtgg gagcttcagg ggaagttctc acaacatgac gccttcgtcg
      241 atgcctgtta cggttacatg gacgacgtcg agcagttcga gtgggactgc tactcagcat
      301 acaatgatgc tgatgttgag cttgccctcg cccgactcca cctcgacacc gtccttaagg
      361 ctcctaaagt taccggctgg cccgtcccta caagacctga cggtgcgcca accacgacgg
      421 aggtccccaa atacccgact ctgcacgaac tcgcagagaa gatccgcaca agcgtgcgac
      481 gagagcgggt atttcaagct gcaggtgaag cgtctggtga taaagatgca gaacctgagt
      541 ctgaagtgcc agaggggtgg cacgttgacg tctggaacag gttccaagac gaggagcgaa
      601 cctactggga aaattactac gccaacccca tcgagttggc gccgcaggtt atacctgtca
      661 acccaggggt cgctgaacct cccatgccca agccgatata caccgagaga gtcgccttta
      721 cggaggacga gctcttcaaa gccgaggctc ggctcaccag ggcaaaatgc tcatactcgt
      781 ccaccattga agacatcaag gaccagtatg aagaagagaa gggagagggc tacttcggcc
      841 gctttttaa cacgtttgaa cagcgaatgc attatgttaa gagagcgcgg agccgtagag
```

```
 901 ccaagacgga ccagctgtgt cacaaggttc aaggtaaact cagtcaggtt gctgaattac
 961 ctgatttcta tgagctgtgt accgtcagag aagtggaaac cggtgagttc cacactgtga
1021 tggatgaagg ggaggaaatc aaacgtccca ttgtcaaagt ctcccgttcc atcaagccgg
1081 aatgccggcg agatgctcaa tcttacatcc gcaagtacat cagatccaaa aacaaccggg
1141 ttggtgcgga tgagatagga gtggccacca tcaatcgata tgtcgcgcag ttcgctgacg
1201 acatgaaact cgacatggcc tcctctgagt ttctcgctcg taccgcgctt accatcgttc
1261 ctgtgataac caagcaagag atgatgcagg caatggtcat ccacagtccc gcggcgagga
1321 aggcgcgcgc ggacctggcc gctcttgagg gccaggattt ttagaggggc tactgaccgc
1381 atccggcttt gaatcccctt ttagtatttt gggattgccg gaaatcgtgg tgcggtcagg
1441 atgcttacct aggaaggtta agagtaggat taattacctg tcccagtttt ccctaggtct
1501 agactatcgt atacccaatc cttcatttca caacgccctt gtggctgttg agcggcgggt
1561 tttcaccgtc ggcaagggtg acgatattgt gcgccctcca aaaccccgga ggaacatttt
1621 tgaagagcgc ttgggttact tccgcgacaa gattgtcgct gatgtcgggc cgctacgggc
1681 atgttccgtg gcacaattgg tctccaccta caaatcgagc aagaggaggc agtatgagtt
1741 ggccgcgttc aaacttcgaa agaagccagt atgcaaggaa gacgctgacg ttactgcatt
1801 cctcaaaatg gagaagcact ggatgtgcaa ggcaattgcc cctagattga tctgccccg
1861 aagcaaaaga tacaatattg agcttgggcg ccgtttgaag ctgaacgaaa aacgattcat
1921 gcatgccatt gacaatgtct ttgggtcagc gacggtgctc agtggctacg acaattttaa
1981 gcaggggaga ttgatcgctg gtaagtggaa caaattcagg aatcctgtgg ctattggagt
2041 agacgcctcc cgcttcgacc aacatgtgtc gacggaggcg ttgaagtggg agcacagcat
2101 ctacaacaag gtttttgggg accctctgtt gcgtgacttg ttggactggc aaacagtaaa
2161 caagtgcagt ctttttgttg aagacaagat gttgcgcttc aaggttaagg gccacaggat
2221 gtctggtgat attaatacca gtatggggaa caagctcatt atgtgcggaa tgatgcacaa
2281 ctatttccgt gaacttggag tgaaagctga actttgcaac aatggtgacg attgcgtcat
2341 catttgtgaa cgcaaagacg aaaggaagtt cgacggttta ggaaaatggt tttgggagta
2401 cggattcaac atggctattg agccgcctgt atactccctg gccaagcttg aattttgcca
2461 gtcccgacca gtttgtatca atgggaagta tagaatggtt cgccgtcccg actcaatcgc
2521 gaaggacgcc cataccatgc tcagtatgca gaatgcagag gatgtaaaaa gttttatgtc
```

```
2581 tgctactggc cagtgtggta tgattttaaa ctctggcgtc cccattttgg atgcgtatca
2641 ttctaatttg tatagaggtt cgggctacaa gaaggtatct gagagcttta ttgatcgagt
2701 catatcttat gggacagatg agcgcctcca gggtcgacgg acccgtgttg aggaaccagt
2761 aactatggaa aatcggttga gttactggga tgcttttggg gttgatccgc aaacacaggt
2821 ccttgttgaa cgttatctca acaatttgcg gatcggatgc gagcccctgg gagtgaagat
2881 agtgactcct cttctcacaa gcaccttgct tgaaatacct tattataaac ctctcaattt
2941 agcaccatag attttcattt actagccggc ttctttttag gtttcttagc tagtatacct
3001 attacagttt gtgtgtgcta cgtagcctat attaaaatct cccaacaagt tcgttcaata
3061 gtgaacgagt atggtcgtgc gtagacgtca gccaattaga agaaatatca ggcgacgacg
3121 caatggacct cgcaggtttg cagcaccgcc acgggtggtt gtggtcccag gacggcctcg
3181 aagacgaaga agaaatggaa gaacaaaccc tcgagttaac cgaggcagaa ttaccttctc
3241 ttcgcggcca gctgaggtct tcactttcac agtggacgac ctcaaagccg gatccacggg
3301 ggtcctcaag ttcggaccgg gcctatcaca gtgcgctgcg gtttcagggg gagtactcaa
3361 gtcctaccat caatataaaa tcatcggtct cacgtgcggt tatgtcacga acgccagcag
3421 caccactgcc ggcgcatttg ctctggagat cgacactacc tgttctcgaa gcgcccttga
3481 atcaagaatc atttcattcc ccgtcacgaa gaacacttca aaattcttcc cgccgggggt
3541 cattaatggg cagaattgga tcagctctga cacgaaccaa ttcttcctcc tctatggagg
3601 aaatggatcc aagaccgaga tcgcgggaca gttactcatc aaggtgatga taactttgca
3661 aggtcccaaa taggtagacg cagctccatc accttcaccc aagcctgacc cgaaacccag
3721 tcctcctcca ccttcaccga aacccgcaaa ggaaaggcga tttttcgcct actctggtat
3781 accaaaaacg aagatcaaaa ctaaaggcaa tgacgactcc atcattgcct cctccaattt
3841 ggagcagcag gtcttccggt atatagaggc aaacaatcaa aaagacgtca cgttgaacgc
3901 gcgttggtat tcgacttcca cagtgaagaa caagccaatg attgtattcg acgtgcccgc
3961 tggtgattgg tttgttgatt tcctttgtga gggttacatg cctattgagg caatcggtgg
4021 tagtgaggac cagaagtgga tgggaattgt ggcgtacaac aatgataccg cggacatttg
4081 gtctgtcgga gtgtacgaca atgtctcaat cactgagctc aacataactt cctcttggaa
4141 gcttggacat aaagatttgg agctcaatgg gtgtcatttt catgacggtc aggttgtaga
4201 gagagatagc atcggttcat gtaaagtatc atctaatacc ggtggatctc tctttttggt
```

```
4261 ggcaccatcc attatgaaga cagaaaagta caattactgc gtctcatatg gcgattacac
4321 tgacaaaacc ttggagtttg gttttgtatc catggtgttt gatgagcgtg atggagctaa
4381 caccgccgtt ccgcacatta gaagagagct caagaatgtt aaatatcttc ggccttctcc
4441 tgtgcgtctg agcgacggcg gtgattatat tgatgaagtt cagaaaccga ttgcagcggc
4501 ccccccttct gcaaagcgac cctccagtgc aagacacgtg gtcgcaccgg aacctcaacc
4561 ggaacctgcc ccggaacctc aaccgggagt ctcacagtct cccaagcgtg aacccgcttt
4621 gccggttaat gagccattct ggccaatctc agttattgat agcatacatg ttgcggaagt
4681 caccacatct gatgaatcca agatacgtgt acctttagaa actcgagacc cagatggcaa
4741 tatcctctcc ctacaccctg gcggtttaaa tgccatgggc cgtgacctac agcaattcga
4801 aagggatgct gtttacaaga tgtgggtcga gggacaagca gaggacatac ggcgaaaaca
4861 gattgagact gatgccgcct tagcacgttc tatttctgag aacgactacc gccagataaa
4921 ccaagaaata cgagctgccg agttgcctaa tcaaccgaac tttgtatacc gggacgaccc
4981 tattgtgaag caaaacagca cgtctgattt cattgctgca cgtagggctg atttcgatga
5041 acaaagtgct tcggacttga aatctaatgc atcaactcgg acaattactg ggaatcttgg
5101 cggtggaaat ctgaagaaga aggcgagcga ccttgatgtt gttgaggaca gaatcttgaa
5161 ggcattacca gaaattgatt ataaaccttc agaaatcctc ggcgttaagg ctcggtatca
5221 tggaggatgc ggcaagtgga gagacacctt tgattcatcc atgaattgcc gttgttggat
5281 gccaaccctt gaatggcaac aagttgattt tcaatacaag gggaaagcat ccagaaatga
5341 gggaaagttc atgatctcat ggccaccgta gtgtacacac cttacctagt agcattcata
5401 tttaaatatt gcacatacag aagcaaaatg ccaaagctct ctggtgtacg tcctggtaga
5461 acaggcatct gaagataact caccgcttgc ggtcagctct gtctcagtgt aagttaggcg
5521 gggtctagtc aaccccggtc cgcacgcatc agtagtggta ttaacaaaat agctctgtga
5581 aacaactaaa aattagtgta acttttattt ttcttttctt atagaaaaac ctgccccctt
5641 cacgcgggga ggatggaaaa catgggtatg atggacctag ccagtcctgg tgtgatgaaa
5701 acagcggaga tgaatgcccg tggttgtttt cggggaagcg acgccctgga cgttcgcagc
5761 ttgcttctga gctatacact gcccgtgact gcagcacggt aacgaattgc tggccccacc
5821 tcgatgagac gagaggtggg tcctcgaggt gccgtcacct cgttaaacaa cgcgactgtg
5881 tgtgaactga cacagtcggg cgccaaatcc acttttggtg gtctgtttgg cgttgcccct
```

```
5941 cctgaaatgg ggagggccga ggcactaaga cctcgggcgg tagggcattt gcgcccggat
6001 cgatgcttgt catccatccc gccacctacc accaccggcc cctggttcgt aactaggagt
6061 tgaaggttag ctaaaacctt tgacacgcaa tcaaacgaca tccagaaaag ttgtttgatc
6121 gtttgatcgt gtgtcacaca ccacggcctc tcgaggcttt tggtgtgcta gtggtcctct
6181 cctgcctgag aggaacgcag cgtgaggggg atgctcacgt gggcggccag gccccaactg
6241 gcccctgtgt ctcggacacc tttcacacat gacctagcca agtgtgtgga agtatcccta
6301 ccccaaaggt agggggtag ctagactttt gcgcggtgcc accggaaacg gaagtgcacc
6361 cc
```
