# Supplementary material for: Characterizing the Virome of Apple Orchards Affected by Rapid Decline in the Okanagan and Similkameen Valleys of British Columbia (Canada)
Source: Pathogens. 2022 Oct 25;11(11):1231. doi: 10.3390/pathogens11111231 (PMC9698585; doi:10.3390/pathogens11111231)
Supplement: Supplementary file 1 [file pathogens-11-01231-s001.zip › Text S2.pdf]

Text S2. Annotated sequence of ALV1 isolate BC85 as submitted to NCBI

```
LOCUS       Seq2                    6390 bp    RNA     linear   VRL 19-AUG-
2022
DEFINITION  Apple luteovirus 1 isolate BC85, near complete genome.
ACCESSION   Seq2
VERSION
KEYWORDS    .
SOURCE      Apple luteovirus 1
  ORGANISM  Apple luteovirus 1
            Viruses; Riboviria; Orthornavirae; Kitrinoviricota;
            Tolucaviricetes; Tolivirales; Tombusviridae; Luteovirus.
REFERENCE   1  (bases 1 to 6390)
  AUTHORS   Xiao,H., Hao,W., Storoschuk,G., MacDonald,J. and Sanfacon,H.
  TITLE     Virus prevalence in apple orchards affected by rapid decline and
            identification of a novel highly prevalent ilarvirus in the
            Okanagan valley of British Columbia (Canada)
  JOURNAL   unpublished
REFERENCE   2  (bases 1 to 6390)
  AUTHORS   Xiao,H. and Sanfacon,H.
  TITLE     Direct Submission
  JOURNAL   Submitted (19-AUG-2022) Summerland Research and Development
Centre,
            Agriculture & Agri-Food Canada, 4200 Highway 97, Summerland
            Research and Development Centre, SUMMERLAND, BC V0H 1Z0, Canada
COMMENT     Bankit Comment: ALT EMAIL:huogen@gmail.com
            Bankit Comment: TOTAL # OF SEQS:1
```

```
            ##Assembly-Data-START##
            Assembly Method       :: CLC Genomic Work Bench 20 v. 20.0.4
            Sequencing Technology :: Illumina
            ##Assembly-Data-END##
FEATURES             Location/Qualifiers
     source          1..6390
                     /organism="Apple luteovirus 1"
                     /mol_type="genomic RNA"
                     /isolate="BC85"
                     /host="Malus domestica"
                     /db_xref="taxon:2170544"
                     /country="Canada"
                     /collection_date="16-July-2020"
     gene            89..2949
                     /gene="ORF1-ORF2"
     CDS             join(89..1360,1360..2949)
                     /gene="ORF1-ORF2"
                     /ribosomal_slippage
                     /note="RNA dependent RNA polymerase P1-P2 fusion; -1
                     ribosomal frameshift slippage"
                     /codon_start=1
                     /product="P1-P2 polyprotein"
/translation="MLFDDLICASFKVVKDFISHIYNNLRTVYKKFKVWLWELQGKFT
QHDAFVDACYGYMDDVEKFEWDCYSSYNDADVELALAKMHLDTIRKAPKVTGWPVPTR
PDGAPTTTEVPKNPTLHELAEKIRTSVRRERVFRAAGEASGDKDAEPEADVPEGWHVD
```

```
VWNKFQDEERTYWENYYASPIELAPQVIPVNPGVAEPPMPKPIYTERVAFTEDELFKA
EARLTRAECSYASTIEDIKDAYEEEKGEGYFGRFFNTFEQRMAYVKRARSRRAKTDQL
CHKVQGKLGSFDELPDFYELCTVREVETGEFHTVMDEGEEIKRPIVKISRSIKEDCRR
QAQSYIRKYIKSKNNRVGADEIGVATINRYVAQFADDMKLDIASTEFLARTALTIVPV
ISKQEMMQAMVIHSPAARKARADLAALEGQDFLEGLLTASGFESPFSILGLPEIVVRS
GCLPRKVKSRISYLSQFSLGLDYRIPNPSFHNALVAVERRVFTVGKGDDIVRPPKPRR
NIFEERLGYFRDKIVADVGPLRTCTVAQLVSTYKSSKRRQYELAAFKLRKKPVCKEDA
DVTAFLKMEKHWMCKAIAPRLICPRSKRYNIELGRRLKLNEKRFMHAIDNVFGSATVL
SGYDNFKQGRLIAGKWNKFRNPVAIGVDASRFDQHVSIEALKWEHSIYNKVFGDPLLR
DLLDWQTVNKCSLFVEDKMLRFKVKGHRMSGDINTSMGNKLIMCGMMHNYFRELGVRA
ELCNNGDDCVIICERKDEKKFDGLGKWFWEYGFNMAIEPPVYSLAKLEFCQSRPVCIN
GKYRMVRRPDSIAKDANTMLSMQNAEDVKSFMSATGQCGMILNSGVPILDAYHSNLYR
GSGYKKVSESFIDRVISYGTDERLQGRRTRVEEPVTMENRLSYWDAFGVDPQTQVLVE
                     RYLNNLRIGCEPLGVKIVTPLLTSTLLEIPYFKPLNLAP"
     gene            89..1363
                     /gene="ORF1"
     CDS             89..1363
                     /gene="ORF1"
                     /note="RNA dependent RNA polymerase"
                     /codon_start=1
                     /product="P1"
```

```
/translation="MLFDDLICASFKVVKDFISHIYNNLRTVYKKFKVWLWELQGKFT

QHDAFVDACYGYMDDVEKFEWDCYSSYNDADVELALAKMHLDTIRKAPKVTGWPVPTR

PDGAPTTTEVPKNPTLHELAEKIRTSVRRERVFRAAGEASGDKDAEPEADVPEGWHVD

VWNKFQDEERTYWENYYASPIELAPQVIPVNPGVAEPPMPKPIYTERVAFTEDELFKA

EARLTRAECSYASTIEDIKDAYEEEKGEGYFGRFFNTFEQRMAYVKRARSRRAKTDQL

CHKVQGKLGSFDELPDFYELCTVREVETGEFHTVMDEGEEIKRPIVKISRSIKEDCRR

QAQSYIRKYIKSKNNRVGADEIGVATINRYVAQFADDMKLDIASTEFLARTALTIVPV
                     ISKQEMMQAMVIHSPAARKARADLAALEGQDF"
     gene            225..1019
                     /gene="ORF0"
     CDS             225..1019
                     /gene="ORF0"
                     /codon_start=1
                     /product="putative P0 protein"

/translation="MTPSSTHVMDIWTMSRNSNGTATHHTMMLMLSWHSPRCTSTPSV

RLLRSQAGPSQQDRTVRQPRRRSPRTRLCMNSQRRSGRVSDESGFLELQVKRLVIKMQ

NLKLTCQRGGTSTSGISSKTKSVPTGKTTTPAPSSWRRRLYLSTQGSLNLPCPSRYTL

KESPLRRTNSSRQRHGLPEPSAHMHPLLKTSRMLMKRKRGRATSAAFLTRLSREWLML
                     KERGAVEPRRTSCAIKFKVNSVRLTNYLISMSCVPSEKWKLGNSTL"
     gene            2946..3083
                     /gene="ORF3a"
     CDS             2946..3083
```

```
                     /gene="ORF3a"
                     /note="viral movement; alternative translation
initiation
                     at an AUA"
                     /codon_start=1
                     /transl_except=(pos:2946..2948,aa:Met)
                     /product="P3a protein"

/translation="MDYHLLAGFFLGFLASIPITVCVCYVAYIKISQQVRSIVNEYGR
                     A"
     gene            3070..5370
                     /gene="ORF3-ORF5"
     CDS             3070..5370
                     /gene="ORF3-ORF5"
                     /note="read-through stop codon in ORF3"
                     /codon_start=1
                     /transl_except=(pos:3670..3672,aa:OTHER)
                     /product="P3-P5 polyprotein"

/translation="MVVRRRQPVRRNIRRRRNGPRRFAAPPRVVVVPGRPRRRRRNGR

TNPRANRGRITFSSRPAEVFTFTVDDLKAGSTGVLKFGPGLSQCAAVSGGVLKSYHQY

KIIGLTCGYVTNASSTTAGAFALEIDTTCSRSALESRIISFPVTKNTSKFFPPGVING

QNWVSSDTNQFFLLYGGNGSKTEIAGQLLIKVMITLQGPKXVDAAPSPSPKPDPKPSP

PPPSPKPAKERRFFAYSGIPKTKIKTKGNDDSIIASSNLEQQVFRYIEANYQKDVTLN

ARWYSTSTAKNKPMIVFDVPAGDWFVDFLCEGYMPIEAIGGSEDQKWMGIVAYNNDTA

DIWSVGVYDNVSITELNITSSWKLGHKDLELNGCHFHDGQVVERDSIGSCKVSSNTGG
```

```
SLFLVAPSIMKTEKYNYCVSYGDYTDKTLEFGFVSMVFDERDGANTAVPHIRRELKNV

KYLRPSPVRLSDGGDYIDEVQKPIAAAPLSAKRPPNARSMVAPEPKPVPAPEPQPEVS

QSPTREPAVPTNEPFWPISVIDSIHVAEVTTSDESKIRVPLETRDPDGNILALHPGGL

NAMGRDLQQFERDAVYKMWVEGQAEDIRRKQIETDAALARSISENDYRQINQEILATE

LPNQPNFVYRDDPIVKQNSTSDFIAARRADFDEQSISDLKSNASTRTITGNLGGGKLK

KKASDLDVVEDRILKAVPGIDYKPSEILGVKARYHGGCGKWKDTFDSSMNCRCWMPTL
                     EWQQVDFQYKGKASRNEGKSMISWPP"
     gene            3070..3672
                     /gene="ORF3"
     CDS             3070..3672
                     /gene="ORF3"
                     /codon_start=1
                     /product="coat protein"

/translation="MVVRRRQPVRRNIRRRRNGPRRFAAPPRVVVVPGRPRRRRRNGR

TNPRANRGRITFSSRPAEVFTFTVDDLKAGSTGVLKFGPGLSQCAAVSGGVLKSYHQY

KIIGLTCGYVTNASSTTAGAFALEIDTTCSRSALESRIISFPVTKNTSKFFPPGVING
                     QNWVSSDTNQFFLLYGGNGSKTEIAGQLLIKVMITLQGPK"
     gene            3122..3646
                     /gene="ORF4"
     CDS             3122..3646
                     /gene="ORF4"
                     /codon_start=1
                     /product="movement protein"
```

```
/translation="MDLAGLQHRHGWLWSQEGLEDEEEMEEQTLELTEAELPSLRGQL

RSSLSQWTTSKPDPRGSSSSDRAYHSALRFQGEYSSPTINIKSSVSRAVMSRTPAAPL

PAHLLWRSTLPVLEAPLNQESFHSPSRRTLQSSSRRGSLMGRIGSALTRTNSSSSMEE
                     MDPRPRSRDSYSSR"
     gene            4472..4654
                     /gene="ORF5a"
     CDS             4472..4654
                     /gene="ORF5a"
                     /codon_start=1
                     /product="putative P5a protein"

/translation="MKFRNRLQRPPFLQSDLQMLDPWSHRNLNRYLLRNLNRKSHSLQ
                     RGSPLCRLTSHSGQSQ"
     gene            5358..5471
                     /gene="ORF6"
     CDS             5358..5471
                     /gene="ORF6"
                     /codon_start=1
                     /product="putative P6 protein"
                     /translation="MATVVYTPYLVAFIFYYCTYRSEMPKLSDVRPGRTGI"
     gene            5653..5802
                     /gene="ORF7"
     CDS             5653..5802
                     /gene="ORF7"
                     /codon_start=1
                     /product="putative P7 protein"
```

```
/translation="MGNMGMMDLASPGVMKTAEMNARGCFRGSDALDVRSLLLSYTLP
                     VTAAR"
     gene            5948..6064
                     /gene="ORF8"
     CDS             5948..6064
                     /gene="ORF8"
                     /codon_start=1
                     /product="putative P8 protein"
                     /translation="MGRAEALRPRAVGHLRLDRCLSSIPPPTTTGPWFVTRS"
BASE COUNT     1679 a   1535 c   1649 g   1527 t
ORIGIN
        1 cgatcatcac acccaaagtc cttgctttat ctgtcagcgg cttagtatac cgtctcagag
       61 tttataattg aaacccaggc ccgtcaagat gttgttcgac gacctcatct gcgccagttt
      121 caaggttgtt aaagatttca tctcacacat ctacaacaac ctccgcaccg tgtacaagaa
      181 gtttaaggtg tggctgtggg aactccaagg taagttcaca cagcatgacg ccttcgtcga
      241 cgcatgttat ggatatatgg acgatgtcga gaaattcgaa tgggactgct actcatcata
      301 caatgatgct gatgttgagt tggcactcgc caagatgcac ctcgacacca tccgtaaggc
      361 tcctaaggtc acaggctggc ccgtcccaac aagaccggac ggtgcgccaa ccacgacgga
      421 ggtccccaag aacccgactc tgcatgaact cgcagagaag atccggacga gtgtccgacg
      481 agagcgggtt tttagagctg caggtgaagc gtctggtgat aaagatgcag aacctgaagc
      541 tgacgtgcca gaggggtggc acgtcgacgt ctggaataag ttccaagacg aagagcgtac
      601 ctactgggaa aactactacg ccagccccat cgagctggcg ccgcaggtta tacctgtcaa
      661 cccaggggtc gctgaacctc ccatgcccaa gccgatatac actgaaagag tcgcctttac
      721 ggaggacgaa ctcttcaagg cagaggcacg gcttacccga gccgagtgct catatgcatc
      781 cactattgaa gacatcaagg atgcttatga agaggaaaag ggggagggct acttcggccg
      841 ctttttaac acgtttgagc agagaatggc ttatgttaaa agagcgcgga gccgtagagc
      901 caagacggac cagctgtgcc ataaagttca aggtaaactc ggttcgtttg acgaattacc
```

961 tgatttctat gagctgtgta ccgtcagaga agtggaaact ggggaattcc acactgtgat

1021 ggatgaaggg gaggagatca agcgtcctat tgtcaaaatc tcccgttcca tcaaggaaga

1081 ctgccgtcgc caggcacaat cctacatccg caagtacatc aaatctaaga acaacagagt

1141 tggtgcggat gagataggtg ttgcaactat caaccggtat gttgcccagt ttgctgatga

1201 catgaaactg gatatcgcct caacggagtt cctggcgcgc acagccctta ccattgttcc

1261 tgtaataagc aagcaagaaa tgatgcaggc aatggtcatc cacagtcccg cggcgaggaa

1321 ggcgcgcgcg gacctggccg ctcttgaggg ccaggatttt tagaggggct actgaccgca

1381 tccggctttg aatcccttt tagtattttg ggattgccgg aaatcgtggt gcggtcagga

1441 tgcttaccta ggaaggttaa gagtaggatt agttacttat ctcagttttc cctaggtcta

1501 gactatcgta tacccaatcc ttcatttcac aacgccctcg tggctgttga gcggcgagtt

1561 ttcaccgtcg gcaagggtga cgatatagtg cgccctccaa aaccccggag gaacattttt

1621 gaagagcgct tgggttactt ccgcgacaag attgtcgctg atgtcgggcc actgcggaca

1681 tgtaccgtag cgcaattggt ctccacctac aaatcgagca agaggaggca gtatgagttg

1741 gccgcgttca aacttcgaaa gaagcctgta tgcaaggaag atgctgacgt caccgctttc

1801 ctcaaaatgg agaagcactg gatgtgcaag gcaattgccc cgagattgat ctgcccccga

1861 agcaaaaggt acaacatcga gcttgggcgc cgtttgaagc tgaatgaaaa gcgatttatg

1921 catgccattg acaatgtctt tggatcagca acggtgctca gtggctacga taatttcaaa

1981 caggggagat tgatcgctgg taagtggaac aaattcagga atcctgtggc tattggagta

2041 gacgcctctc gcttcgacca acatgtgtcg attgaggcgt tgaagtggga gcatagcatc

2101 tacaacaagg tctttgggga ccctctgttg cgcgacttgt tggactggca aacagtaaac

2161 aagtgcagtc tttttgttga agacaagatg ctacgcttca aggttaaggg ccacaggatg

2221 tctggtgata ttaacaccag tatggggaac aagctcatta tgtgcggaat gatgcacaac

2281 tatttccgtg aacttggagt gagagctgaa ctttgcaaca atggtgacga ctgcgtcatc

2341 atttgtgaac gcaaagatga aaagaagttc gacggattag ggaaatggtt ttgggagtac

2401 ggctttaaca tggctattga gccacctgta tactccctag ccaagcttga gttttgccag

2461 tcccgaccag tttgtataaa cgggaagtat agaatggttc gccgtcctga ttcaattgca

2521 aaggacgcca ataccatgct cagtatgcag aatgcagagg atgtgaagag tttcatgtct

2581 gctactggcc agtgtggtat gattttaaat tctggcgtcc ccattttgga tgcataccat

2641 tctaatttat atagaggttc gggctacaag aaggtatctg agagcttcat tgatagagtc

2701 atatcttatg ggacagatga gcgcctccag ggtcgacgga ctcgtgttga ggaaccagta

2761 actatggaaa atcggttgag ttactgggat gcttttgggg ttgatccgca gacacaggtc

2821 cttgttgaac gttatctcaa caatctgcgg atcggatgcg aacccctggg agtgaagata

2881 gtgactcctc ttctcacaag cacactgctt gaaatacctt attttaaacc tcttaattta

2941 gcaccataga ttaccattta ctagcaggct tttcttagg tttcttagct agtataccta

3001 ttacagtttg tgtgtgctac gtagcctaca ttaaaatctc ccaacaagtt cgttcaatag

3061 tgaacgagta tggtcgtgcg tagacgtcag ccagtcagaa ggaatatcag gcgacgacgc

3121 aatggacctc gcaggtttgc agcaccgcca cgggtggttg tggtcccagg aaggcctcga

3181 agacgaagaa gaaatggaag aacaaaccct cgagctaacc gaggcagaat taccttctct

3241 tcgcggccag ctgaggtctt cactttcaca gtggacgacc tcaaagccgg atccacgggg

3301 gtcctcaagt tcggaccggg cctatcacag tgcgctgcgg tttcaggggg agtactcaag

3361 tcctaccatc aatataaaat catcggtctc acgtgcggtt atgtcacgaa cgccagcagc

3421 accactgccg gcgcatttgc tctggagatc gacactacct gttctcgaag cgccccttgaa

3481 tcaagaatca tttcattccc cgtcacgaag aacacttcaa agttcttccc gccgggggtc

3541 attaatgggc agaattgggt cagctctgac acgaaccaat tcttcctcct ctatggagga

3601 aatggatcca agaccgagat cgcgggacag ttactcatca aggtgatgat aactttgcaa

3661 ggccccaaat aggtagacgc agctccatca ccttcaccca aacctgaccc gaaacccagt

3721 cctcctccac cttcaccgaa gcccgcaaag gaaaggcgat ttttcgccta ctctggtata

3781 ccaaagacga agatcaaaac taaaggcaat gacgactcca tcattgcctc ctccaatttg

3841 gagcagcagg tcttccggta tatagaggca aactatcaga aagacgtcac gttgaacgcg

3901 cgttggtatt cgacttccac agcgaagaac aagccaatga tcgtattcga cgtgcccgct

3961 ggtgattggt ttgttgattt cctttgtgag ggttatatgc ctattgaggc aataggtggt

4021 agtgaggacc agaagtggat gggaattgta gcgtacaaca atgataccgc ggatatttgg

4081 tccgttggag tgtacgacaa tgtctcaatc actgagctca acataacttc ctcttggaag

4141 cttggtcata aagatttaga gctcaatggg tgtcattttc atgacggtca ggttgtagag

4201 agagatagta tcggttcatg taaagtatca tccaataccg gtggatctct cttcttggtg

4261 gcaccatcca ttatgaagac agaaaagtac aactactgtg tctcatatgg cgattacact

```
4321 gacaaaacct tggagtttgg ttttgtatct atggtgtttg atgagcgtga tggagctaac
4381 accgctgttc cgcacattag aagagagctc aagaatgtca aatatcttcg gccttctcct
4441 gtgcgtctga gcgacggcgg tgattatatt gatgaagttc agaaaccgat tgcagcggcc
4501 cccctttctg caaagcgacc tccaaatgct agatccatgg tcgcaccgga acctaaaccg
4561 gtacctgctc cggaacctca accggaagtc tcacagtctc caacgcggga gcccgctgtg
4621 ccgactaacg agccattctg gccaatctca gtaattgata gcatacatgt tgcggaagtc
4681 accacatctg atgaatccaa aatacgtgta cctttagaaa ctcgagaccc agatggcaat
4741 atcctcgccc tccaccccgg cggtttgaat gccatgggtc gtgacctaca gcagtttgaa
4801 agagatgctg tttacaaaat gtgggtcgag ggacaagcag aggatatacg gcgaaagcaa
4861 attgagactg atgccgcctt agcacgttct atctctgaga acgattaccg ccagataaac
4921 caggaaatct tggccaccga gttgcccaat caaccgaact ttgtataccg ggacgaccct
4981 attgtgaagc agaacagcac gtctgatttc attgctgcgc gtagggctga tttcgatgaa
5041 caaagtattt cggatttgaa atccaatgcg tcaactcgga caatcactgg gaatctcggc
5101 ggaggtaaac taaagaagaa ggcgagcgac cttgacgtag ttgaggaccg gatattgaag
5161 gcagttcctg gaatagatta caagccttcc gaaattctcg gcgtcaaggc acggtatcat
5221 ggagggtgcg gcaagtggaa agacaccttt gactcgtcca tgaattgccg ttgttggatg
5281 ccgacccttg agtggcaaca agttgatttc caatataagg ggaaagcatc cagaaatgag
5341 ggaaagtcta tgatctcatg gccaccgtag tgtacacacc ttacctagta gcattcatat
5401 tttattattg cacatacaga agcgaaatgc caaagctctc tgatgtacgt cctggtagaa
5461 caggcatctg aagataactc accgcttgcg gtcagctctg tctcagtgta agttaggcgg
5521 ggtctagtca accccggtcc gtacgcatca gtagtggtat taacaaaata gctctgtgaa
5581 acaactaaaa attagcgtaa cttctgtttt tgctttcctt atagaaaaac ctgcccctt
5641 cacgcgggga ggatgggaaa tatgggtatg atggacttag ccagtcctgg tgtgatgaaa
5701 acagcggaga tgaatgcccg tggttgtttt cggggaagcg acgccctgga cgttcgcagc
5761 ttgcttctga gctatacact gcccgtgact gcagcacggt aacgaattgc tggccccacc
5821 tcgatgagac gagaggtggg tcctcgaggt gccgtcacct cgttaaacaa cgcgactgtg
5881 tgtgaaacct gacacagtcg ggcgtcaaac ccacttcggt ggattgtttg gcgtcgcccc
5941 tcctgaaatg gggagggccg aggcactaag acctcgggcg gtagggcatt tgcgcctgga
```

```
6001 tcgatgcttg tcatccatcc cgccacctac caccaccggc ccctggttcg taactaggag

6061 ttgaaggtta actaaaacct ttgacacaca atcaaacgac atccagaaaa gttgtttgat

6121 catttgatcg tgtgtcacat accacggcct ctcgaggccc ttggtgtgct agtggtcctc

6181 tcctgactga gaggaacgca gcgtgaggga gatgctcacg tgggcggcca ggccccaact

6241 ggcccctgtg tctcggacac ctttcacaca tgacctagcc aagtgtgtgg aagtatccct

6301 accccaaagg tagggggta gctagacttt tgcgcggtgc caccggaaac ggaattttgc

6361 gcggtgccac cggaaacgga agtgcacccc
```
