# Supplementary material for: Characterizing the Virome of Apple Orchards Affected by Rapid Decline in the Okanagan and Similkameen Valleys of British Columbia (Canada)
Source: Pathogens. 2022 Oct 25;11(11):1231. doi: 10.3390/pathogens11111231 (PMC9698585; doi:10.3390/pathogens11111231)
Supplement: Supplementary file 1 [file pathogens-11-01231-s001.zip › Text S3.pdf]

Text S3. Annotated sequence of ALV1 isolate BC134 as submitted to NCBI

```
LOCUS       Seq3                    6389 bp    RNA     linear   VRL 19-AUG-
2022
DEFINITION  Apple luteovirus 1 isolate BC134, near complete genome.
ACCESSION   Seq3
VERSION
KEYWORDS    .
SOURCE      Apple luteovirus 1
  ORGANISM  Apple luteovirus 1
            Viruses; Riboviria; Orthornavirae; Kitrinoviricota;
            Tolucaviricetes; Tolivirales; Tombusviridae; Luteovirus.
REFERENCE   1  (bases 1 to 6389)
  AUTHORS   Xiao,H., Hao,W., Storoschuk,G., MacDonald,J. and Sanfacon,H.
  TITLE     Virus prevalence in apple orchards affected by rapid decline and
            identification of a novel highly prevalent ilarvirus in the
            Okanagan valley of British Columbia (Canada)
  JOURNAL   unpublished
REFERENCE   2  (bases 1 to 6389)
  AUTHORS   Xiao,H. and Sanfacon,H.
  TITLE     Direct Submission
  JOURNAL   Submitted (19-AUG-2022) Summerland Research and Development
Centre,
            Agriculture & Agri-Food Canada, 4200 Highway 97, Summerland
            Research and Development Centre, SUMMERLAND, BC V0H 1Z0, Canada
COMMENT     Bankit Comment: ALT EMAIL:huogen@gmail.com
            Bankit Comment: TOTAL # OF SEQS:1
```

```
            ##Assembly-Data-START##
            Assembly Method       :: CLC Genomic Work Bench 20 v. 20.0.4
            Sequencing Technology :: Illumina
            ##Assembly-Data-END##
FEATURES             Location/Qualifiers
     source          1..6389
                     /organism="Apple luteovirus 1"
                     /mol_type="genomic RNA"
                     /isolate="BC134"
                     /host="Malus domestica"
                     /db_xref="taxon:2170544"
                     /country="Canada"
                     /collection_date="12-August-2020"
     gene            90..2950
                     /gene="ORF1-ORF2"
     CDS             join(90..1361,1361..2950)
                     /gene="ORF1-ORF2"
                     /ribosomal_slippage
                     /note="RNA dependent RNA polymerase P1-P2 fusion; -1
                     ribosomal frameshift slippage"
                     /codon_start=1
                     /product="P1-P2 polyprotein"
/translation="MLFDDLICASFKVVKDFISHIYNNLRTVYKKFKVWLWELQGKFS
QHDAFVDACYGYMDDVEQFEWDCYSAYNDADVELALARLHLDTVLKAPKVTGWPVPTR
PDGAPTTTEVPKYPTLHELAERIRTSVRRERVFQAAGEASGDKDAEPESEVPEGWHVD
```

```
VWNKFQDEERTYWENYYANPIELAPQVIPVNPGVAEPPMPKPIYTERVAFTEDELFKA

EARLTRAKCSYSSTVEDIKDQYEEEKGEGYFGRFFNTFEQRMHYVKRARSRRAKTDQL

CHKVQGKLSQVAELPDFYELCTVREVETGEFHTVMDEGEEIKRPIVKVSRSIKPECRR

DAQSYIRKYIRSKNNRVGADEIGVATINRYVAQFADDMKLDMASSEFLARTALTIVPV

ITKQEMMQAMVIHSPAARKARADLAALEGQDFLEGLLTASGFESPFSILGLPEIVVRS

GCLPRKVKSRISYLSQFSLGLDYRVPNPSFHNALVAVERRVFTVGKGDDIVRPPKPRR

NIFEERLGYFRDKIVADVGPLRTCTVAQLVSTYKSSKRRQYELAAFKLRKKPVCKEDA

DVTAFLKMEKHWMCKAIAPRLICPRSKRYNIELGRRLKLNEKRFMHAIDNVFGSATVL

SGYDNFKQGRLIAGKWNKFRNPVAIGVDASRFDQHVSTEALKWEHSIYNKVFGDPLLR

DLLDWQTVNKCSLFVEDKMLRFKVKGHRMSGDINTSMGNKLIMCGMMHNYFRELGVKA

ELCNNGDDCVIICERKDERKFDGLGKWFWEYGFNMAIEPPVYSLAKLEFCQSRPVCIN

GKYRMVRRPDSIAKDANTMLSMQNAEDVKSFMSATGQCGMILNSGVPILDAYHSNLYR

GSGYKKVSESFIDRVISYGTDERLQGRRTRVEEPVTMENRLSYWDAFGVDPQTQVLVE
                     RYLNNLRIGCEPLGVKIVTPLLTSTLLEIPYYKPLNLAP"
     gene            90..1364
                     /gene="ORF1"
     CDS             90..1364
                     /gene="ORF1"
                     /note="RNA dependent RNA polymerase"
                     /codon_start=1
                     /product="P1"
```

```
/translation="MLFDDLICASFKVVKDFISHIYNNLRTVYKKFKVWLWELQGKFS

QHDAFVDACYGYMDDVEQFEWDCYSAYNDADVELALARLHLDTVLKAPKVTGWPVPTR

PDGAPTTTEVPKYPTLHELAERIRTSVRRERVFQAAGEASGDKDAEPESEVPEGWHVD

VWNKFQDEERTYWENYYANPIELAPQVIPVNPGVAEPPMPKPIYTERVAFTEDELFKA

EARLTRAKCSYSSTVEDIKDQYEEEKGEGYFGRFFNTFEQRMHYVKRARSRRAKTDQL

CHKVQGKLSQVAELPDFYELCTVREVETGEFHTVMDEGEEIKRPIVKVSRSIKPECRR

DAQSYIRKYIRSKNNRVGADEIGVATINRYVAQFADDMKLDMASSEFLARTALTIVPV
                     ITKQEMMQAMVIHSPAARKARADLAALEGQDF"
     gene            226..1020
                     /gene="ORF0"
     CDS             226..1020
                     /gene="ORF0"
                     /codon_start=1
                     /product="putative P0 protein"

/translation="MTPSSMPVTVTWTTSSSSSGIATQHTMMLMLSLPSPDSTSTPSL

RLLKLPAGPSQQDRTVRQPRRRSPNTRRYMNSQRGSAQACEESGYFKLQVKRLVIKMQ

NLSLKCQRGGTLTSGTSSKTRSEPTGKITTPTPSSWRRRLYLSTQGSLNLPCPSRYTP

RESPLRRTSSSKLRLGSPGQNVHTRPPLKISRTSMKKRRERATSAVFLTRLNSECIML
                     KERGAVEPRRTSYATRFKVNSARLLNYLISMSCVPSERWKLVSSTL"
     gene            2947..3084
                     /gene="ORF3a"
     CDS             2947..3084
```

```
                     /gene="ORF3a"

                     /note="viral movement; alternative translation
initiation

                     at an AUA"

                     /codon_start=1

                     /transl_except=(pos:2947..2949,aa:Met)

                     /product="P3a protein"

/translation="MDFHLLAGFFLGFLASIPITVCVCYVAYIKISQQVRSIVNEYGR

                     A"

     gene            3071..5371

                     /gene="ORF3-ORF5"

     CDS             3071..5371

                     /gene="ORF3-ORF5"

                     /note="read-through stop codon in ORF3"

                     /codon_start=1

                     /transl_except=(pos:3671..3673,aa:OTHER)

                     /product="P3-P5 polyprotein"

/translation="MVVRRRQPVRRNIRRRRNGPRRFAAPPRVVVVPGRPRRRRRNGR

TNPRANRGRITFSSRPAEVFTFTVDDLKAGSTGVLKFGPGLSQCAAVSGGVLKSYHQY

KIIGLTCGYVTNASSTTAGAFALEIDTTCSRSALESRIISFPVTKNTSKFFPPGVING

QNWISSDTNQFFLLYGGNGSKTEIAGQLLIKVMITLQGPKXVDAAPSPSPKPDPRPGP

PPPSPKPAKERRFFAYSGIPKTKIKTKGNDDSIIASSNLEQQVFRYIEANNQKDVTLN

ARWYSTSTVKNKPMIVFDVPAGDWFVDFLCEGYMPIEAIGGSEDQKWMGIVAYNNDTA

DIWSVGVYDNVSITELNITSSWKLGHKDLELNGCHFHDGQVVERDSIGSCKVSSNTGG
```

```
SLFLVAPSIMKTEKYNYCVSYGDYTDKTLEFGFVSMVFDERDGANTAVPHIRRELKNV

KYLRPSPVRLSDGGDYIDEVQKPIAAAPLPAKRPPNARSMVAPEPKPVPAPEPQPEVS

QSPKREPAVPTNEPFWPISVIDSIHVAEVTTSDESKIRVPLETRDPDGNILALHPGGL

NAMGRDLQQFERDAVYKMWVEGQAEDIRRKQIETDAALARSISENDYRQINQEILATE

LPNQPNFVYRDDPIVKQNSTSDFIAARRADFDEQSISDLKSNASTRTITGNLGGGKLK

KKASDLDVVEDQILKAVPGIDYKPSEILGVKARYHGGCGKWKDTFDSSMNCRCWMPTL
                     EWQQVDFQYKGKASRNEGKSMISWPP"
     gene            3071..3673
                     /gene="ORF3"
     CDS             3071..3673
                     /gene="ORF3"
                     /codon_start=1
                     /product="coat protein"

/translation="MVVRRRQPVRRNIRRRRNGPRRFAAPPRVVVVPGRPRRRRRNGR

TNPRANRGRITFSSRPAEVFTFTVDDLKAGSTGVLKFGPGLSQCAAVSGGVLKSYHQY

KIIGLTCGYVTNASSTTAGAFALEIDTTCSRSALESRIISFPVTKNTSKFFPPGVING
                     QNWISSDTNQFFLLYGGNGSKTEIAGQLLIKVMITLQGPK"
     gene            3123..3647
                     /gene="ORF4"
     CDS             3123..3647
                     /gene="ORF4"
                     /codon_start=1
                     /product="movement protein"
```

```
/translation="MDLAGLQHRHGWLWSQDGLEDEEEMEEQTLELTEAELPSLRGQL

RSSLSQWTTSKPDPRGSSNSDRAYHSALRFQGEYSSPTINIKSSVSRAVMSRTPAAPL

PAHLLWRSTLPVLEAPLNQESFHSPSRRTLQSSSRRGSLMGRIGSALTRTNSSSSMEE
                     MDPRPRSRDSYSSR"
     gene            4473..4655
                     /gene="ORF5a"
     CDS             4473..4655
                     /gene="ORF5a"
                     /codon_start=1
                     /product="putative P5a protein"

/translation="MKFRNRLQRPPFLQSDLQMLDPWSHRNLNRYLLRNLNRKSHSLQ
                     SGSPLCRLTSHSGQSQ"
     gene            5654..5803
                     /gene="ORF7"
     CDS             5654..5803
                     /gene="ORF7"
                     /codon_start=1
                     /product="putative P7 protein"

/translation="MENMGMMDLASPGVMKTAEMNARGCFRGSDALDVRSLLLSYTLP
                     VTAAR"
     gene            5949..6065
                     /gene="ORF8"
     CDS             5949..6065
                     /gene="ORF8"
                     /codon_start=1
```

```
                     /product="putative P8 protein"
                     /translation="MGRAEALRPRAVGHLRLDRCLSSIPPPTTTGPWFVTRS"
BASE COUNT     1661 a   1553 c   1653 g   1522 t
ORIGIN
        1 cgatcatcac aaaccaaagc ccctcgcttt atctgtcagc ggtttagttt gccgtctcag
       61 agtttcagtg tgaaaccaag cccgtcaaga tgttgtttga cgacctcatc tgcgctagtt
      121 tcaaggttgt aaaagatttc atctctcaca tctacaacaa cctccgcacc gtctacaaaa
      181 aatttaaggt gtggctgtgg gagcttcagg ggaagttctc acaacatgac gccttcgtcg
      241 atgcctgtta cggttacatg gacgacgtcg agcagttcga gtgggattgc tactcagcat
      301 acaatgatgc tgatgttgag cttgccctcg cccgactcca cctcgacacc gtccttaagg
      361 ctcctaaagt taccggctgg cccgtcccaa caagaccgga cggtgcgcca accacgacgg
      421 aggtccccaa atacccgacg ctacatgaac tcgcagagag gatccgcaca agcgtgcgaa
      481 gagagcgggt atttcaagct gcaggtgaag cgtctggtga taaagatgca gaacctgagt
      541 ctgaagtgcc agaggggtgg cacgttgacg tctggaacaa gttccaagac gaggagcgaa
      601 cctactggga aaattactac gccaacccca tcgagttggc gccgcaggtt atacctgtca
      661 acccaggggt cgctgaacct cccatgccca agccgatata caccgagaga gtcgccttta
      721 cggaggacga gctcttcaaa gctgaggctc ggctcaccag ggcaaaatgt tcatactcgt
      781 ccaccgttga agatatcaag gaccagtatg aagaagagaa gggagagggc tacttcggcc
      841 gtttttttaa cacgtttgaa cagcgaatgc attatgttaa aagagcgcgg agccgtagag
      901 ccaagacgga ccagctatgc cacaaggttc aaggtaaact cagccaggtt gctgaattac
      961 ctgatttcta tgagctgtgt accgtcagag aggtggaaac tggtgagttc cacactgtga
     1021 tggatgaagg ggaggagatt aaacgtccaa ttgtcaaagt ctcccgttcc atcaagccgg
     1081 aatgccggcg agatgctcag tcttacatcc gcaagtacat cagatccaag aataatcggg
     1141 ttggtgcgga tgagatagga gtggccacca tcaatcggta tgtcgcgcag ttcgctgacg
     1201 acatgaaact cgacatggcc tcctctgagt ttctcgcgcg caccgcgctt accatcgtgc
     1261 ctgtgataac caagcaagag atgatgcagg cgatggtcat ccacagtccc gcggcgagga
     1321 aggcgcgcgc ggacctggcc gcccttgagg gccaggattt ttagaggggc tactgaccgc
     1381 atccggcttt gaatcccctt ttagtatttt gggattgccg gaaatcgtgg tgcggtcagg
```

1441 atgcttacct aggaaagtta agagtaggat tagctatttg tcccagtttt ccctaggttt

1501 agactatcgt gtacccaatc cttcatttca caacgccctc gtggctgttg agcggcgggt

1561 tttcaccgtc ggcaagggtg acgatatagt gcgccctcca aaaccccgga ggaacatttt

1621 tgaagagcgc ttgggttact tccgcgacaa gattgtcgct gatgtcgggc cgctacggac

1681 atgtaccgta gcgcaactgg tctccaccta caaatcgagt aagaggaggc agtatgagtt

1741 ggccgcgttc aaacttcgaa agaagcctgt gtgcaaggaa gacgctgacg ttactgcttt

1801 cctcaaaatg gagaagcact ggatgtgcaa ggcaatcgcc ccaagattga tctgcccccg

1861 aagcaaaagg tacaatatcg agcttgggcg ccgtttgaag ctgaatgaaa agcgatttat

1921 gcatgccatt gacaacgtct ttggatcagc aacggtgctc agtggttacg ataacttcaa

1981 gcaggggaga ttgatcgctg gtaagtggaa caaattcaga aatcctgtgg ctattggagt

2041 agacgcctct cgtttcgatc aacatgtgtc gactgaggcg ttgaagtggg agcatagcat

2101 ctacaacaag gtttttgggg accctctgtt gcgcgacttg ttggactggc aaacagtaaa

2161 caagtgcagt ctttttgttg aagacaagat gctacgcttc aaggttaagg gccataggat

2221 gtctggtgat attaatacca gtatggggaa caagcttatt atgtgcggaa tgatgcacaa

2281 ctatttccgt gaacttggag tgaaagctga actttgtaac aatggtgacg attgcgtcat

2341 catttgtgaa cgcaaagacg aaaggaagtt cgacggatta ggaaagtggt tttgggagta

2401 cggattcaac atggctattg agcctcctgt atactccctg gccaagcttg aattttgcca

2461 gtcccgacca gtttgtatta atgggaagta tagaatggtt cgccgtcctg actccatcgc

2521 gaaggacgcc aataccatgc tcagtatgca gaatgcagaa gatgtgaaaa gtttcatgtc

2581 tgctactggc cagtgtggta tgattttgaa ttctggcgtc cccattttgg acgcgtacca

2641 ttctaatttg tatagaggtt cgggctacaa gaaggtatct gagagcttca ttgatagagt

2701 catatcttat gggacagatg agcgcctcca gggtcgacgg acccgtgttg aggaaccagt

2761 aactatggaa aatcggttga gttactggga tgcttttggg gttgatccgc aaacacaggt

2821 ccttgttgaa cgttatctca acaatttgcg gatcggatgc gagcccctgg gagtgaagat

2881 agtgactcct cttctcacaa gcaccttgct tgaaatacct tattataaac ctctcaattt

2941 agcaccatag attttcattt actagccggc tttttcttag gtttcttagc tagtatacct

3001 attactgttt gtgtgtgcta cgtagcctat attaaaatct cccagcaagt ccgttcaata

3061 gtgaacgagt atggtcgtgc gtagacgtca gccagtcaga agaaatatca ggcgacgacg

```
3121 caatggacct cgcaggtttg cagcaccgcc acgggtggtt gtggtcccag gacggcctcg
3181 aagacgaaga agaaatggaa gaacaaaccc tcgagctaac cgaggcagaa ttaccttctc
3241 ttcgcggcca gctgaggtct tcactttcac agtggacgac ctcaaagccg gatccacggg
3301 ggtcctcaaa ttcggaccgg gcctatcaca gtgcgctgcg gtttcagggg gagtactcaa
3361 gtcctaccat caatataaaa tcatcggtct cacgtgcggt tatgtcacga acgccagcag
3421 caccactgcc ggcgcatttg ctctggagat cgacactacc tgttctcgaa gcgcccttga
3481 atcaagaatc atttcattcc ccgtcacgaa gaacacttca aagttcttcc cgccgggggt
3541 cattaatggg cagaattgga tcagctctga cacgaaccaa ttcttcctcc tctatggagg
3601 aaatggatcc aagaccgaga tcgcgggaca gttactcatc aaggtgatga taactttgca
3661 aggtcccaaa taggtagacg cagctccatc gccctcaccg aaacctgacc ctagacccgg
3721 tcctcctcca ccttcaccga aacccgcaaa ggaaaggcga tttttcgcct actctggtat
3781 accaaagacg aagatcaaaa ctaaaggcaa tgacgactcc atcattgcct cctccaattt
3841 ggagcagcag gtcttccggt atatagaggc aaacaatcag aaagacgtca cgttgaacgc
3901 gcgttggtat tcgacttcca ctgtgaagaa caagccaatg atcgtattcg acgtgcccgc
3961 aggtgattgg tttgttgatt tcctttgtga gggttatatg cctattgagg caataggtgg
4021 cagtgaggac cagaagtgga tgggaattgt agcgtacaac aatgataccg cggatatttg
4081 gtccgtcgga gtgtacgaca atgtctcaat cactgagctc aacataactt cctcttggaa
4141 gcttggtcat aaagatttag agctcaatgg gtgtcatttc catgacggtc aggttgtaga
4201 gagagatagc atcggttcat gtaaagtatc atccaatacc ggtggatccc tcttcttggt
4261 ggcaccatcc attatgaaga cagaaaagta caactactgt gtctcatatg gcgattacac
4321 tgacaaaacc ttggagtttg gttttgtatc tatggtgttt gatgagcgtg atggagctaa
4381 caccgctgtt ccgcacatta gaagagagct caagaatgtt aaatatcttc ggccttctcc
4441 tgtgcgtctg agcgacggcg gtgattatat tgatgaagtt cagaaaccga ttgcagcggc
4501 ccccccttcct gcaaagcgac ctccaaatgc tagatccatg gtcgcaccgg aacctaaacc
4561 ggtacctgct ccggaacctc aaccggaagt ctcacagtct ccaaagcggg agcccgctgt
4621 gccgactaac gagccattct ggccaatctc agtaattgat agcatacatg ttgcggaagt
4681 caccacatct gatgaatcca aaatacgtgt acctttagaa actcgagacc cagatggcaa
4741 tatcctcgcc ctccaccccg gcggtttgaa tgccatgggt cgtgacctcc aacagtttga
```

```
4801 aagagatgct gtttacaaga tgtgggtcga gggacaagca gaggatatac ggcgaaagca
4861 aattgagact gatgccgcct tagcacgttc tatctctgag aacgattacc gccagataaa
4921 ccaggaaatc ttggccaccg agttgcccaa tcaaccgaac tttgtatacc gggacgaccc
4981 tattgtgaag cagaacagca cgtctgattt cattgccgcg cgtagggctg atttcgatga
5041 acaaagtatt tcggacttga aatccaatgc gtcaactcgg acaatcactg ggaatctcgg
5101 cggaggcaaa ctaaagaaga aggcgagcga ccttgacgtt gttgaggacc agatattgaa
5161 ggcagttcct ggaattgatt acaagccttc agaaatcctc ggcgtcaagg cacggtatca
5221 tggaggatgc ggcaagtgga aagacacctt tgactcttcc atgaattgcc gttgttggat
5281 gccgaccctt gagtggcaac aagttgattt ccaatataag gggaaagcat ccagaaatga
5341 gggaaagtct atgatctcat ggccaccgta gtgtacacac cttacctagt agcattcata
5401 ttttaatatt gcacatacag aagcgaaatg ccaaagctct ctgatgtacg tcctggtaga
5461 acaggcatct gaagataact caccgcttgc ggtcagctct gtctcagtgt aagttaggcg
5521 gggtctagtc aaccccggtc cgtacgcatc agtagtggta ttaacaaaat agctctgtga
5581 aacaactaaa aattagcgta acttctgttt ttgctttcct tatagaaaaa cctgccccct
5641 tcacgcgggg aggatggaaa atatgggtat gatggactta gccagtcctg gtgtgatgaa
5701 aacagcggag atgaatgccc gtggttgttt tcggggaagc gacgccctgg acgttcgcag
5761 cttgcttctg agctatacac tgcccgtgac tgcagcacgg taacgaattg ctggccccac
5821 ctcgatgaga cgagaggtgg gtcctcgagg tgccgtcacc tcgttaaaca acgcgactgt
5881 gtgtgaaacc tgacacagtc gggcgtcaaa cccacttcgg tggattgttt ggcgtcgccc
5941 ctcctgaaat ggggagggcc gaggcactaa gacctcgggc ggtagggcat ttgcgcctgg
6001 atcgatgctt gtcatccatc ccgccaccta ccaccaccgg cccctggttc gtaactagga
6061 gttgaaggtt aactaaaacc tttgacacac aatcaaacga cacccagaaa agttgtttga
6121 tcatttgatc gtgtgtcaca taccacggcc tctcgaggcc cttggtgtgc tagtggtcct
6181 ctcctgactg agaggaacgc agcgtgaggg ggatgctcac gtgggcggcc aggccccaac
6241 tggcccctgt gtctcggaca cctttcacac atgacctagc caagtgtgtg gaagtatccc
6301 taccccaaag gtaggggggt agctagactt ttgcgcggtg ccaccggaaa cggaagtgca
6361 ccccacccca tgaaaacagc ggagatgaa
```
