# Supplementary material for: Characterizing the Virome of Apple Orchards Affected by Rapid Decline in the Okanagan and Similkameen Valleys of British Columbia (Canada)
Source: Pathogens. 2022 Oct 25;11(11):1231. doi: 10.3390/pathogens11111231 (PMC9698585; doi:10.3390/pathogens11111231)
Supplement: Supplementary file 1 [file pathogens-11-01231-s001.zip › Text S4.pdf]

Text S4. Annotated sequence of AIV2 RNA1, 2 and 3 as submitted to NCBI

>AIV2 RNA1 (156A-RNA1-Id-4) consisting of 3456 bases
GGTATTGTCAACACTTATTTGTGTGACGACCCGGATTAATCAGTAAAACTGAGTATATACCCGTTAGCAC
TCATGGAAACTATTGTTGATCATAGTTCTTCTGCGCTCCGTATGGACGATCTGTTGAATGATGTCATACG
TCGTAATGCGTCCCAAACCACCACAGATGTTGGTCGGTTGGTCTCAGATGCCGCTGTGCAAGTGATCCGC
CGTCAGGTGGATCTCACACCAGCGAAGCCGCTGAACATTTCGTTCGCGTTGACGCCTGAAGAGCAGATGG
CTCTCAGGCGTGATTTTCCCGGTCGAGAGTTTCAGTTTAAGAATTCTGCATATTCCTCACATTCCTTTGC
TGCCGCACATCGTGTGTGTGAGACGGATTTTATCTATTCTCGCTTTCAGACCGAAGCCACTACCATTATT
GATATTGGTGGAAATTTTGCCACTCATGCGAAGATGGGTAGATGTAATGTACACTCGTGTTGCCCAATTT
TGGACGTTCGAGATGGTGCACGTTATACCGACAGATTTGCCTCTATTGCTGGTTCGCTTGAGAAGCAACC
AGATAGGGAGTTGCCCTTGAATTTCTGTTCGAAGAAGTTTGAAGAATGTGAGGTTTCAGCCCCTTGGGCT
ATGGCCATACACTCTATTTCAGACATTCCCATCACTACTGTCGTTGAATCATGTTTTAGACGTGGTGTCA
AGAAGTTGATTGCATCGATCATGATGGATGAATATATGCTTATCGCGACTGAAGGCTTTATTCCGCGAAT
AAATGTTCAGTGGAAGATTGAGAGGGACATTGATCCAGACACTGGTGAAGCCATTGGACCTCGTTGGGTC
TATTTCCATTTTCTTGATGCTCCTGGCCTGTCTTATAGGCATAATTATGATGTGCTTATGCAATATATGC
ACTGTAACCAAGTCATAATTAATGGGAAAGCCGCGTATCGTGTTGAGCGCGTTGCCAATCTATCAGGAGT
CTACATTGTAGAGATTACCCTGGCTGCGACTGCAGGTCTTGAAAGACTGGATTTGAAGCCCACGCACGAC
GTTTCATGTGCATGGTTGACGTCTTTGAGGAAAAAGACTCTCATTCGGGTAGCGATTCCCCAGATGCGTA
ATTCCTGGGAGATAAAGTATGTCATTGCTGATACAGATTTTGTGCGACGTGTGGCTGAAGTTTCTTTCCG
CCAATACAAACCAGATACTCCTTTGGAGCAGTTGGTTCAGTCTGTAGCGACAATGATTTCCTCTGCATCA
AATCATTGTATTATTAATGGAGTGACTATGCAAACAGGTACACCGTTGGCTATTGAGGATTATGTTCCAA
TGGCCGTGACCTTTGTGGCGTTTGCAAAATCTCGATACAAGAGTATAAAATCCTCCTTTGATATGGTGAG
ACAGAGAGGTACTTGTGTGGCTGATCCTAATGATAATTACGATTATGAGAAGGATGGGTCGGACAAGAAA
TCTTTTCTTAATCATGTGATACCCATGAAAAATACCATCAAATCCCTTTTTTTCCCTATGAAGGCAAAAA
TTTCCGACAACTCGATGATGATTTATAATTCGTCTATCCTTTCCGATGTTGTTGATGAGTTGAAATCTGC
CATGGGATGGGAAATTTGGGATACCGATGATGCAGTTATAAAATCTATGCCTTCCTTTTATAAGATGGAA
GATGTTTATACTGTTGTTTCTGATCATCATTGCCTATCCACCACTCTATGTGTTGATTGGTGGTTAGAAG
GTCTTTATGATGATCATGAAACGTTGCGCAGGGTTCATAAGAAGAAACTGCAAGAAGAGGAAGCGAGAAA
AACTAAAGTTGAAAATGCCCTCTTAAAAATTGCCGAGGTTTTGGAGAAACCCAATGTTCCTGATGGACTG
GCAAAGTTAACTACTTTACCCATCATTTCCTCCTTATTGGAGAAGAAGGAAGAAACCATTGTGACAAAGC
CTCGATGCAGGGATTCAGAATTGCCCCATATAAATCCCTTTGCTGATTCTATCAAAGAAGCCATGAGCTA
TTACCACGAACTTGAGGTGGTTGCTGCCCGAAATCTTAGAGGAGTTGGTGATTACCTAGGATGGCGGACG
AAACAGAACTATGCCAGCGTTTGGGGAGCTGATGAGAGTCGATGTGTATTAGAACCGATGAAACGGCGGT
TCTTTCGTTCCGACCCACGTGTTTCCGTGCCTAGATTTGAGAGAGGCATGACGCAGGATGGTTGGGTTAC
TCTGGCTTACGAATCTATTGATGATAATGTAGTCACAGAAACCACCTGGAAGTCAATTTCCAAATATGCG
GTGATTTTGTTCGATTCATCTTGCATGTTTGATGCCACATCTCGTATAATGCCGGCCATGGAAAAGTCTC
TTACCATGATGCCGAAGTTTTCAGTAGTCGAGGTGGAAGATGGTGTAGCCGGTTGTGGAAAAACAACATC
TCTACTCAAGCAGGCTAAGCCTGATTCAGATCTGTTGTTATCGGCTAATCGTGAAACGGCAAAGGATGCT
CGTGACAGTGGTGTTATACCTGATCCTCTAAAATACCGAGTGCGAACTGTAGATTCATACTTGATGTTGA
AGAAATGGTTTACCGCTGAGAGATTGTTAGTAGATGAATGTTTCCTACTACATTCTGGAGTCATTTATGC
TGCCGCAACTGTGAGTCAGGTGAAACAAGTTATCGCCTTTGGTGATACGAAACAAATTCCATTTATTTCA
CGTATTCCCACGGTAAAACTTCGTCACGCTAGGGTGCAAGGGAAATTGAATCCTAAAACTATTACCTATC
GATGTCCAAGAGATGTTACTGCGATTTTGAGTGAGAGATTTTACCATACTAAGGTAAAAACCTTTAATCC
CACAGAAACATCTGTTGAAATCATTCCCATAAATTCGAAATTGGAAGTTCCGATAGAGAAGGATACAATG
TACATTGCCCACCTACGTGCTGACAAGGACTCCCTAATGCAATTGGGGGTTCCCAAGGCGCGAGATCATT
CCACGCATGAGGCTCAGGGAAAAACAGCCAAGGATGTTATTTTGGTGCGGTTTAGTAAAACAGGAAATTT
GCTTTATTCCGGCAAGATGCCAGATGCTGGGAATTCGCACAATCTTGTTGGCTTGTCCCGACACACTCGG
TCGTTGAGATATTACACCGTGTGTGCTGATGATCCAGATGATGAAATAGCATCTGGAATTCGATGGTCAA
AAACTTTAGACCATCAAGTTCTCGCTACATATCGTGCGGGAAGTTAGTGACTCTACATGAGTCCTAGTGA
AGGATCCTCCGGGATGTGCTCAGCACCTAGCCTAAGCTAAGTTCATATGCCCACCTATGCTGCTCCGGGT

GGATGCTTATTTGTTTATGAATGCCTATGATTGAAATATCATAGATGCCTAAATTTTTCTCTCTTGAGAA
AAATTTAGATGCCTCCTAGGAGACGC

>ORF number 1 in reading frame 1 on the direct strand extends from base 73 to base 3267.

ATGGAAACTATTGTTGATCATAGTTCTTCTGCGCTCCGTATGGACGATCTGTTGAATGAT
GTCATACGTCGTAATGCGTCCCAAACCACCACAGATGTTGGTCGGTTGGTCTCAGATGCC
GCTGTGCAAGTGATCCGCCGTCAGGTGGATCTCACACCAGCGAAGCCGCTGAACATTTCG
TTCGCGTTGACGCCTGAAGAGCAGATGGCTCTCAGGCGTGATTTTCCCGGTCGAGAGTTT
CAGTTTAAGAATTCTGCATATTCCTCACATTCCTTTGCTGCCGCACATCGTGTGTGTGAG
ACGGATTTTATCTATTCTCGCTTTCAGACCGAAGCCACTACCATTATTGATATTGGTGGA
AATTTTGCCACTCATGCGAAGATGGGTAGATGTAATGTACACTCGTGTTGCCCAATTTTG
GACGTTCGAGATGGTGCACGTTATACCGACAGATTTGCCTCTATTGCTGGTTCGCTTGAG
AAGCAACCAGATAGGGAGTTGCCCTTGAATTTCTGTTCGAAGAAGTTTGAAGAATGTGAG
GTTTCAGCCCCTTGGGCTATGGCCATACACTCTATTTCAGACATTCCCATCACTACTGTC
GTTGAATCATGTTTTAGACGTGGTGTCAAGAAGTTGATTGCATCGATCATGATGGATGAA
TATATGCTTATCGCGACTGAAGGCTTTATTCCGCGAATAAATGTTCAGTGGAAGATTGAG
AGGGACATTGATCCAGACACTGGTGAAGCCATTGGACCTCGTTGGGTCTATTTCCATTTT
CTTGATGCTCCTGGCCTGTCTTATAGGCATAATTATGATGTGCTTATGCAATATATGCAC
TGTAACCAAGTCATAATTAATGGGAAAGCCGCGTATCGTGTTGAGCGCGTTGCCAATCTA
TCAGGAGTCTACATTGTAGAGATTACCCTGGCTGCGACTGCAGGTCTTGAAAGACTGGAT
TTGAAGCCCACGCACGACGTTTCATGTGCATGGTTGACGTCTTTGAGGAAAAAGACTCTC
ATTCGGGTAGCGATTCCCCAGATGCGTAATTCCTGGGAGATAAAGTATGTCATTGCTGAT
ACAGATTTTGTGCGACGTGTGGCTGAAGTTTCTTTCCGCCAATACAAACCAGATACTCCT
TTGGAGCAGTTGGTTCAGTCTGTAGCGACAATGATTTCCTCTGCATCAAATCATTGTATT
ATTAATGGAGTGACTATGCAAACAGGTACACCGTTGGCTATTGAGGATTATGTTCCAATG
GCCGTGACCTTTGTGGCGTTTGCAAAATCTCGATACAAGAGTATAAAATCCTCCTTTGAT
ATGGTGAGACAGAGAGGTACTTGTGTGGCTGATCCTAATGATAATTACGATTATGAGAAG
GATGGGTCGGACAAGAAATCTTTTCTTAATCATGTGATACCCATGAAAAATACCATCAAA
TCCCTTTTTTTCCCTATGAAGGCAAAAATTTCCGACAACTCGATGATGATTTATAATTCG
TCTATCCTTTCCGATGTTGTTGATGAGTTGAAATCTGCCATGGGATGGGAAATTTGGGAT
ACCGATGATGCAGTTATAAAATCTATGCCTTCCTTTTATAAGATGGAAGATGTTTATACT
GTTGTTTCTGATCATCATTGCCTATCCACCACTCTATGTGTTGATTGGTGGTTAGAAGGT
CTTTATGATGATCATGAAACGTTGCGCAGGGTTCATAAGAAGAAACTGCAAGAAGAGGAA
GCGAGAAAAACTAAAGTTGAAAATGCCCTCTTAAAAATTGCCGAGGTTTTGGAGAAACCC
AATGTTCCTGATGGACTGGCAAAGTTAACTACTTTACCCATCATTTCCTCCTTATTGGAG
AAGAAGGAAGAAACCATTGTGACAAAGCCTCGATGCAGGGATTCAGAATTGCCCCATATA
AATCCCTTTGCTGATTCTATCAAAGAAGCCATGAGCTATTACCACGAACTTGAGGTGGTT
GCTGCCCGAAATCTTAGAGGAGTTGGTGATTACCTAGGATGGCGGACGAAACAGAACTAT
GCCAGCGTTTGGGGAGCTGATGAGAGTCGATGTGTATTAGAACCGATGAAACGGCGGTTC
TTTCGTTCCGACCCACGTGTTTCCGTGCCTAGATTTGAGAGAGGCATGACGCAGGATGGT
TGGGTTACTCTGGCTTACGAATCTATTGATGATAATGTAGTCACAGAAACCACCTGGAAG
TCAATTTCCAAATATGCGGTGATTTTGTTCGATTCATCTTGCATGTTTGATGCCACATCT
CGTATAATGCCGGCCATGGAAAAGTCTCTTACCATGATGCCGAAGTTTTCAGTAGTCGAG
GTGGAAGATGGTGTAGCCGGTTGTGGAAAAACAACATCTCTACTCAAGCAGGCTAAGCCT
GATTCAGATCTGTTGTTATCGGCTAATCGTGAAACGGCAAAGGATGCTCGTGACAGTGGT
GTTATACCTGATCCTCTAAAATACCGAGTGCGAACTGTAGATTCATACTTGATGTTGAAG
AAATGGTTTACCGCTGAGAGATTGTTAGTAGATGAATGTTTCCTACTACATTCTGGAGTC
ATTTATGCTGCCGCAACTGTGAGTCAGGTGAAACAAGTTATCGCCTTTGGTGATACGAAA
CAAATTCCATTTATTTCACGTATTCCCACGGTAAAACTTCGTCACGCTAGGGTGCAAGGG

AAATTGAATCCTAAAACTATTACCTATCGATGTCCAAGAGATGTTACTGCGATTTTGAGT
GAGAGATTTTACCATACTAAGGTAAAAACCTTTAATCCCACAGAAACATCTGTTGAAATC
ATTCCCATAAATTCGAAATTGGAAGTTCCGATAGAGAAGGATACAATGTACATTGCCCAC
CTACGTGCTGACAAGGACTCCCTAATGCAATTGGGGGTTCCCAAGGCGCGAGATCATTCC
ACGCATGAGGCTCAGGGAAAAACAGCCAAGGATGTTATTTTGGTGCGGTTTAGTAAAACA
GGAAATTTGCTTTATTCCGGCAAGATGCCAGATGCTGGGAATTCGCACAATCTTGTTGGC
TTGTCCCGACACACTCGGTCGTTGAGATATTACACCGTGTGTGCTGATGATCCAGATGAT
GAAATAGCATCTGGAATTCGATGGTCAAAAACTTTAGACCATCAAGTTCTCGCTACATAT
CGTGCGGGAAGTTAG

>Translation of ORF number 1 in reading frame 1 on the direct strand.
/product="putative viral replicase", 1064aa
METIVDHSSSALRMDDLLNDVIRRNASQTTTDVGRLVSDAAVQVIRRQVDLTPAKPLNIS
FALTPEEQMALRRDFPGREFQFKNSAYSSHSFAAAHRVCETDFIYSRFQTEATTIIDIGG
NFATHAKMGRCNVHSCCPILDVRDGARYTDRFASIAGSLEKQPDRELPLNFCSKKFEECE
VSAPWAMAIHSISDIPITTVVESCFRRGVKKLIASIMMDEYMLIATEGFIPRINVQWKIE
RDIDPDTGEAIGPRWVYFHFLDAPGLSYRHNYDVLMQYMHCNQVIINGKAAYRVERVANL
SGVYIVEITLAATAGLERLDLKPTHDVSCAWLTSLRKKTLIRVAIPQMRNSWEIKYVIAD
TDFVRRVAEVSFRQYKPDTPLEQLVQSVATMISSASNHCIINGVTMQTGTPLAIEDYVPM
AVTFVAFAKSRYKSIKSSFDMVRQRGTCVADPNDNYDYEKDGSDKKSFLNHVIPMKNTIK
SLFFPMKAKISDNSMMIYNSSILSDVVDELKSAMGWEIWDTDDAVIKSMPSFYKMEDVYT
VVSDHHCLSTTLCVDWWLEGLYDDHETLRRVHKKKLQEEEARKTKVENALLKIAEVLEKP
NVPDGLAKLTTLPIISSLLEKKEETIVTKPRCRDSELPHINPFADSIKEAMSYYHELEVV
AARNLRGVGDYLGWRTKQNYASVWGADESRCVLEPMKRRFFRSDPRVSVPRFERGMTQDG
WVTLAYESIDDNVVTETTWKSISKYAVILFDSSCMFDATSRIMPAMEKSLTMMPKFSVVE
VEDGVAGCGKTTSLLKQAKPDSDLLLSANRETAKDARDSGVIPDPLKYRVRTVDSYLMLK
KWFTAERLLVDECFLLHSGVIYAAATVSQVKQVIAFGDTKQIPFISRIPTVKLRHARVQG
KLNPKTITYRCPRDVTAILSERFYHTKVKTFNPTETSVEIIPINSKLEVPIEKDTMYIAH
LRADKDSLMQLGVPKARDHSTHEAQGKTAKDVILVRFSKTGNLLYSGKMPDAGNSHNLVG
LSRHTRSLRYYTVCADDPDDEIASGIRWSKTLDHQVLATYRAGS*

>AIV2 RNA2 (156A_RNA2_II_2) consisting of 2913 bases
GGTATTGTCAACACTTATTTGTGTGACGACCCGGATTAATCAGTAAAACTGAGTATATACCCGTTAGATT
TACTCTTTACCATGGCTGCTGTTCACATGATGTTGTCATCTTTGTGTAAGTTGACCGATTTGGATTCCGC
TTTCTCTGTTTATTCCTCTTTGGGGTTGGACATTGCAAGCGATGCGACTATCCTCCATCATTTACGTTTA
TGGCTTTTGGCCAAGGTTTTCTTTCCGGTTTGGAAGTTTGAACTCCGTGCAGACACTATTCTCTTTAAGT
TAGAAGATGTGTTGTGTCCAGAGGTTCCCATTCCCACCGATGTGGAGGTTTGTTCATGGACTGATGCAGA
TTATGTTCCGTATGTACCACCGAGTTTTGTTGATGAATTTGGTGAAGTTCCTGAGGAGGCGTTTTTACCC
TTAGAAGAAAGAGAGGCCATTTATAAACCCACTTTTCTCACTCACGAGCATTGGGCTAGTGAGTCTTCGG
GGTCTTCTTTTCTCGACGACATTGAAGTTTTTGCTGACGAGAAACACACCGATGATTGTGTTCAAGATGT
GTGCACTAATATGCCCCCAGATTATCAGATTATGTGGGATGAAGGTGCAGTGGATGCCGTCTGGTCAAGT
CGTTTCGAATGCGAAGAACCACCTAAACCACAATTTCAGGCTGTTTTGACAGATAAAGTTTGTGATCCAG
TGGTTATTCAAGATGCCATAAATGACATATTCCCGTTTCATCATGAGATGGATGATAGATATTTCCAAAC
AATGGTAGAAACTGATGACATTTCCTTGGAAGTCTCGAAGTGTTGGATAGATGCCTCAAATTTCCGAGAT
TTCACAAAAGGTCAAAGCTCATATGCAGAGCCAGTCTACCAATCTGGTGCCACTAGCCGGCGTGTAAACA
CTCAGCGTGAAACACTGCTGGCCGTGAAGAAGAGAAATATGAATATTCCCGATTTACAATCTGTTTTTGA
TTTGGATGCGGAAGTCAACTTGTGTACGAAGCGATTCCTTACGCATGTTATTGACATGCCACGTTTGCGG
AGGTTACCACCTATGATGGGATGTGAAATTGATTTTTTCACGGCGTATTTGGCAGGGAAAAACCCCCCCA
TTAAGGAATACCAGGGACCCTTATCACTGGTTTCGTTGGATAAATACCTTCACATGGTGAAGACGATTAT
TAAGCCAGTAGAAGATAATTCTCTGAAGTTTGAAAGGCCTCTTTGTGCTACGATCACCTATCATAAAAAG

GGGATTGTAATGCAGTCATCTCCATTGTTTTTGAGTGCAATGTCGAGATTGTTCTATGTTTTGAAATCAA
AAATACATATACCGAGTGGTAAATGGCATCAATTGTTTACTCTCGATGCGGCCCATTTTGATGCGGCCAA
GTGGTTTAAGGAAGTGGACTTTTCAAAGTTTGATAAATCTCAAGGGGAACTGCATCATTTGGTGCAGAGA
AATATATTCCATGCTTTGAAATTACCTCCAGAATTTGTGGAGATGTGGTTCACTTCTCATGAACGGTCCC
ATATAATTGATCGTGAAACAGGAGTGGGGTTTTCCGTTGATTTTCAACGACGTACCGGAGATGCAAATAC
CTACCTGGGGAATACGTTGGTGAATTTGATATGTTTGGCCAGGGTTTATAACCTTAGCGATCCCAATATC
ACCTTTGTTATTGCTTCGGGAGATGATTCACTCATCGGGAGTTTAGTGGAACTTCCAAGAGACTCCGAAG
ATCTTTTCTCTACTCTATTCAATTTTGAAGCAAAATTTCCTCACAATCAGCCTTTCATTTGCTCAAAGTT
TTTGGTTTCCGTAGATTTGAAAGGGGGTGGTCGAGAGGTTATTGCTGTTCCAAATCCAGCAAAGCTTCTT
ATTCGTATGGGCCGTCGGGATTGCCAATTCCAAGCCATGGAAGATGTGTTTACCTCTTGGCTTGATGTGA
TATATTATTTCCGAGATGCTAGAGTGTGTGAGCGAGTTGCCGATTTATGTGCTTACCGCCAAACTCGTAG
ACCCTCGATGTATTTACTGAGTGCATTGTTAAGTTTGCCTAGTTGCTTTGCAAATTTTAAGAAATTTGCT
TCTGTCTGTTATCATTTAACAGGCAATGATTGTCTGAAGTTAAAAGCAACTAAGAGTTCCCAAAATGGAT
GCCAAAACAATTCCTCAATGCCGACACATGTTAGTCGAAGAGGTGGAACAGTTCGTAAATCCTGTACATT
TCTGCGAGCAAAGGAGATTAGTCGCAATTGCCAGGCGTCAACGTCTGATGTTAAGAATGTCAATTCTCTT
AAGTTTGGTAAGCGTGATAATGTGCATTATCATTGTAACAAACTTTTACCTGTTGAAAGTACAACAGAAG
CAAGCTGCTGTGATGAAAGTGCAAATTCCCACACCGGCTCGTTTACTAGTTCGAGTTCCAGACCTCGCAA
TCGATTTCGAACTAAAGGAGTTCACCAATCCCGCAGTGGTTATTCAAACCATTTTCCGCCAGATAATCGG
CGAAGTTCCTAAGGGATGGTATGGTTTGCAGTCCTGGTCTGATTCCACGTATAATAGCGTGATGTTATCT
TTGAAAAAGTATTCCAGGGCTAAGGTTCATTTTTCAATTCCAGATTCTGATTGGGCTTACACCCTATCGT
TGTCTGATGTGGTTTCTGGGTTAGCACTGCCCAGTTTACCAATTCCTGAGAAGTATCTCAGAAGTGACTC
TTCATGAGTCCTAGTGAAGGATCCTCCGGGATGTGCTCAGCACCTAGCCTAAGCTAAGTTCATATGCCCA
CCTATGCTGCTCCGGGTGGATGTTTATTTGTTTATGAATGCCTATGATTGAAATATCATAGATGCCTAAA
TTTTTCTCTCTTGAGAAAAATTTAGATGCCTCCTAGGAGACGC

>ORF number 1 in reading frame 1 on the direct strand extends from
base 82 to base 2532.
ATGGCTGCTGTTCACATGATGTTGTCATCTTTGTGTAAGTTGACCGATTTGGATTCCGCT
TTCTCTGTTTATTCCTCTTTGGGGTTGGACATTGCAAGCGATGCGACTATCCTCCATCAT
TTACGTTTATGGCTTTTGGCCAAGGTTTTCTTTCCGGTTTGGAAGTTTGAACTCCGTGCA
GACACTATTCTCTTTAAGTTAGAAGATGTGTTGTGTCCAGAGGTTCCCATTCCCACCGAT
GTGGAGGTTTGTTCATGGACTGATGCAGATTATGTTCCGTATGTACCACCGAGTTTTGTT
GATGAATTTGGTGAAGTTCCTGAGGAGGCGTTTTTACCCTTAGAAGAAAGAGAGGCCATT
TATAAACCCACTTTTCTCACTCACGAGCATTGGGCTAGTGAGTCTTCGGGGTCTTCTTTT
CTCGACGACATTGAAGTTTTTGCTGACGAGAAACACACCGATGATTGTGTTCAAGATGTG
TGCACTAATATGCCCCCAGATTATCAGATTATGTGGGATGAAGGTGCAGTGGATGCCGTC
TGGTCAAGTCGTTTCGAATGCGAAGAACCACCTAAACCACAATTTCAGGCTGTTTTGACA
GATAAAGTTTGTGATCCAGTGGTTATTCAAGATGCCATAAATGACATATTCCCGTTTCAT
CATGAGATGGATGATAGATATTTCCAAACAATGGTAGAAACTGATGACATTTCCTTGGAA
GTCTCGAAGTGTTGGATAGATGCCTCAAATTTCCGAGATTTCACAAAAGGTCAAAGCTCA
TATGCAGAGCCAGTCTACCAATCTGGTGCCACTAGCCGGCGTGTAAACACTCAGCGTGAA
ACACTGCTGGCCGTGAAGAAGAGAAATATGAATATTCCCGATTTACAATCTGTTTTTGAT
TTGGATGCGGAAGTCAACTTGTGTACGAAGCGATTCCTTACGCATGTTATTGACATGCCA
CGTTTGCGGAGGTTACCACCTATGATGGGATGTGAAATTGATTTTTTCACGGCGTATTTG
GCAGGGAAAAACCCCCCCATTAAGGAATACCAGGGACCCTTATCACTGGTTTCGTTGGAT
AAATACCTTCACATGGTGAAGACGATTATTAAGCCAGTAGAAGATAATTCTCTGAAGTTT
GAAAGGCCTCTTTGTGCTACGATCACCTATCATAAAAAGGGGATTGTAATGCAGTCATCT
CCATTGTTTTTGAGTGCAATGTCGAGATTGTTCTATGTTTTGAAATCAAAAATACATATA
CCGAGTGGTAAATGGCATCAATTGTTTACTCTCGATGCGGCCCATTTTGATGCGGCCAAG
TGGTTTAAGGAAGTGGACTTTTCAAAGTTTGATAAATCTCAAGGGGAACTGCATCATTTG
GTGCAGAGAAATATATTCCATGCTTTGAAATTACCTCCAGAATTTGTGGAGATGTGGTTC
ACTTCTCATGAACGGTCCCATATAATTGATCGTGAAACAGGAGTGGGGTTTTCCGTTGAT

TTTCAACGACGTACCGGAGATGCAAATACCTACCTGGGGAATACGTTGGTGAATTTGATA
TGTTTGGCCAGGGTTTATAACCTTAGCGATCCCAATATCACCTTTGTTATTGCTTCGGGA
GATGATTCACTCATCGGGAGTTTAGTGGAACTTCCAAGAGACTCCGAAGATCTTTTCTCT
ACTCTATTCAATTTTGAAGCAAAATTTCCTCACAATCAGCCTTTCATTTGCTCAAAGTTT
TTGGTTTCCGTAGATTTGAAAGGGGGTGGTCGAGAGGTTATTGCTGTTCCAAATCCAGCA
AAGCTTCTTATTCGTATGGGCCGTCGGGATTGCCAATTCCAAGCCATGGAAGATGTGTTT
ACCTCTTGGCTTGATGTGATATATTATTTCCGAGATGCTAGAGTGTGTGAGCGAGTTGCC
GATTTATGTGCTTACCGCCAAACTCGTAGACCCTCGATGTATTTACTGAGTGCATTGTTA
AGTTTGCCTAGTTGCTTTGCAAATTTTAAGAAATTTGCTTCTGTCTGTTATCATTTAACA
GGCAATGATTGTCTGAAGTTAAAAGCAACTAAGAGTTCCCAAAATGGATGCCAAAACAAT
TCCTCAATGCCGACACATGTTAGTCGAAGAGGTGGAACAGTTCGTAAATCCTGTACATTT
CTGCGAGCAAAGGAGATTAGTCGCAATTGCCAGGCGTCAACGTCTGATGTTAAGAATGTC
AATTCTCTTAAGTTTGGTAAGCGTGATAATGTGCATTATCATTGTAACAAACTTTTACCT
GTTGAAAGTACAACAGAAGCAAGCTGCTGTGATGAAAGTGCAAATTCCCACACCGGCTCG
TTTACTAGTTCGAGTTCCAGACCTCGCAATCGATTTCGAACTAAAGGAGTTCACCAATCC
CGCAGTGGTTATTCAAACCATTTTCCGCCAGATAATCGGCGAAGTTCCTAA

>Translation of ORF number 1 in reading frame 1 on the direct strand.
/product="putative viral polymerase" 816aa.
MAAVHMMLSSLCKLTDLDSAFSVYSSLGLDIASDATILHHLRLWLLAKVFFPVWKFELRA
DTILFKLEDVLCPEVPIPTDVEVCSWTDADYVPYVPPSFVDEFGEVPEEAFLPLEEREAI
YKPTFLTHEHWASESSGSSFLDDIEVFADEKHTDDCVQDVCTNMPPDYQIMWDEGAVDAV
WSSRFECEEPPKPQFQAVLTDKVCDPVVIQDAINDIFPFHHEMDDRYFQTMVETDDISLE
VSKCWIDASNFRDFTKGQSSYAEPVYQSGATSRRVNTQRETLLAVKKRNMNIPDLQSVFD
LDAEVNLCTKRFLTHVIDMPRLRRLPPMMGCEIDFFTAYLAGKNPPIKEYQGPLSLVSLD
KYLHMVKTIIKPVEDNSLKFERPLCATITYHKKGIVMQSSPLFLSAMSRLFYVLKSKIHI
PSGKWHQLFTLDAAHFDAAKWFKEVDFSKFDKSQGELHHLVQRNIFHALKLPPEFVEMWF
TSHERSHIIDRETGVGFSVDFQRRTGDANTYLGNTLVNLICLARVYNLSDPNITFVIASG
DDSLIGSLVELPRDSEDLFSTLFNFEAKFPHNQPFICSKFLVSVDLKGGGREVIAVPNPA
KLLIRMGRRDCQFQAMEDVFTSWLDVIYYFRDARVCERVADLCAYRQTRRPSMYLLSALL
SLPSCFANFKKFASVCYHLTGNDCLKLKATKSSQNGCQNNSSMPTHVSRRGGTVRKSCTF
LRAKEISRNCQASTSDVKNVNSLKFGKRDNVHYHCNKLLPVESTTEASCCDESANSHTGS
FTSSSSRPRNRFRTKGVHQSRSGYSNHFPPDNRRSS*

>ORF number 5 in reading frame 2 on the direct strand extends from base 2165 to base 2737.
ATGGATGCCAAAACAATTCCTCAATGCCGACACATGTTAGTCGAAGAGGTGGAACAGTTC
GTAAATCCTGTACATTTCTGCGAGCAAAGGAGATTAGTCGCAATTGCCAGGCGTCAACGT
CTGATGTTAAGAATGTCAATTCTCTTAAGTTTGGTAAGCGTGATAATGTGCATTATCATT
GTAACAAACTTTTACCTGTTGAAAGTACAACAGAAGCAAGCTGCTGTGATGAAAGTGCAA
ATTCCCACACCGGCTCGTTTACTAGTTCGAGTTCCAGACCTCGCAATCGATTTCGAACTA
AAGGAGTTCACCAATCCCGCAGTGGTTATTCAAACCATTTTCCGCCAGATAATCGGCGAA
GTTCCTAAGGGATGGTATGGTTTGCAGTCCTGGTCTGATTCCACGTATAATAGCGTGATG
TTATCTTTGAAAAAGTATTCCAGGGCTAAGGTTCATTTTTCAATTCCAGATTCTGATTGG
GCTTACACCCTATCGTTGTCTGATGTGGTTTCTGGGTTAGCACTGCCCAGTTTACCAATT
CCTGAGAAGTATCTCAGAAGTGACTCTTCATGA

>Translation of ORF number 5 in reading frame 2 on the direct strand.
/product="putative 2b protein" 190aa

MDAKTIPQCRHMLVEEVEQFVNPVHFCEQRRLVAIARRQRLMLRMSILLSLVSVIMCIII
VTNFYLLKVQQKQAAVMKVQIPTPARLLVRVPDLAIDFELKEFTNPAVVIQTIFRQIIGE
VPKGWYGLQSWSDSTYNSVMLSLKKYSRAKVHFSIPDSDWAYTLSLSDVVSGLALPSLPI
PEKYLRSDSS*

>AIV2 RNA3 (156A_RNA3_2)consisting of 2269 bases.

GGTGGTATTCTTGATCTTAAGATACCCTTCTGATTCTGTTGTTTTTCCCATCAACCGAGTTTAGTATGTTAACAAATC
CTTAACAGAGCCTCGTTGGTTTCCAACTCGCTACCTGTTTAATCGATGGACTTCGAGAGGATTTGTTTTGATACTTT
CCGAGACTGGTTTAACAGGTGGAATCCTGGAACATCAGAATCGAAGATAGTGGGTTAAGAGTGCTCATCCTCTTC
CCAACATTCGAATACTCCTGATTTGTGCAGGAAAGCCCCCTTGCTGCGATTTCTCATCGTTGGGGTTCTGTTGAGGA
GTACAGATCAGAAGCGACTCCAAAATCGCTGGAGTTGTGCCCGTGTAGTTTCACGGAAGTCCTAGGTGTTACTAGT
TGATTCCGAGTGAGTTATCCTCGAAGGAATGTCTGGGTTTAAAACTAAAGCAGTCGTCTTGAGTGCTACCGATGAA
GAGTCTCTTTTGGCAGAGATTTCCGGTGCCCTTTCCCGTGCCCAAATGGACATGAAGACTTTCCGCGCTTGTGTTCC
CATAGAGATGAAGAATGATAAGGCCTTTTGTTTCGAACTCTGTGATGAAACGACCCGTTCTTGGATCACCGCATTA
AGAGTCAAGGTGCAGGCGAGGCTTAATATTGATCATCCCCACATTATGTTTTTGTGGGTGCCGCGCATTTTGAAAT
CAACCCATGCCACCGTAGATTTAAAAATTCAATATATTGCTACTGGTGATGTGAAGGTTTTAGGTAAGTTGCCATTG
AATGAGGCGTTCCTTCTCAGTTTTGGATGGGAACGCTCAATTAGGATGAAGGATGCGTATGCTAAGAAAGGCCTT
ATGGTTTTTGCGCAACCTAGTGCCCCGGCAACACCTCCAGGAGCCCCTTTGGGTCGCTGGATTCCTATGTGGGACG
TTTGTCCCACACAGAAGATGCGGTATACCGAAGATGTCAAATCTTCGATGACAAAAGCTCAAGAGATGCGTGTGA
AGACTATTTTGAATGAGCGTACCACACGCAGTCTCTTGCGTTCAGTCATGGCAAATGAATATACTTGCCGTGAACA
AGTGCCGAAGTTTTTGGGCCCAAGTGAGGTCCAATTGTCAGATGACTTGCCGGAGTTTCATGATTTTACTCTTGAA
ATGATCTCCGAAAGAAAACCCGAGAACATTCCCCTTAAGGGTGCTGTTAAGGCCATTGAAATCCCCAATAATTTGG
GAGTTGTTCAGTCTGGAAATGGTGTGTCAGGGGATCGAGCCGTCTCTGCTTCAAAGAGGCAAGAACACAATACCC
TGTAAATGACGTTATTAGTGTTTGTGGGATGGAATCCCTTATTTGGCTCCCCCCAAGCACGCGTTGTGTGGGGGTT
CTTGTCTTGAGAAGCCTTTCCTCTAACTGCGTCACTTTCTTCCATTCTGAAAAATGTCGAACAATGCTATTGAGATTA
ATGGGCAGTGGTATATACCACAATCAGGTCGTCAGCCCAAGTCTGTAAATAATGCCCCCGTTATTCGGGGAAGAC
GGAAACCTACGTCCAGGTCGCAAGCCTGGGCTCAAGGTAACCGTTCCCAGCCACCGGATAGGATGATGTTTGGCG
CGATGCGCTCTTCCCTACCCTCCTGGATTAGTACTCCAGGTGAACAGTGGCATGAAGTGGAAGGGTTATCCTTTCC
AGCTTCCTGGACAACAGGTTCCGTGGCTTCTGCCTCGATGCGCACTGAGTTGGGGAAAATAAGACCCCTGCATGAT
TCCACGAAGGTGTATTCCGTGATGTATGGTTTTACCTGCAAATCTGATGGTTATGCCGGATTTGTGGAAGGGTTCG
ATGTGAACAATCCCACTGGTCCGATTGCTCCTAATCGTGTCCGTGTGAAGGCGGGGAAGTATGCAGCTCGTCAGCT
ACGATGCCCCCCTGGTACCACAATTGCCGATTTGAAGGCCTCGTGGTCTTTTGTTTGGCAATTTGATGCCGCCCCAG
CTACCGCCACTGTGAATCAAATCTCAGTGGTTGGTTTTTGGGTTTCTACCACACCTTTGCCTGGTGTGAAGCCACCA
CCAGATTTTCTGGTGTGTGAAGAGTGACTCTACATGAGTCCTAGTGAAGGATCCTCCGGGATGTGCTCAGCACCTA
GCCTAAGCTAAGTTCATATGCCCACCTATGCTGCTCCGGGTGGATGTTTATTTGTTTATGAATGCCTATGATTGAAA
TATCATAGATGCCTAAATTTTTCTCTCTTGAGAAAAATTTAGATGCCTCCAAGGAGACGC

>ORF number 2 in reading frame 1 on the direct strand extends from base 412 to base 1299.
ATGTCTGGGTTTAAAACTAAAGCAGTCGTCTTGAGTGCTACCGATGAAGAGTCTCTTTTG

GCAGAGATTTCCGGTGCCCTTTCCCGTGCCCAAATGGACATGAAGACTTTCCGCGCTTGT
GTTCCCATAGAGATGAAGAATGATAAGGCCTTTTGTTTCGAACTCTGTGATGAAACGACC
CGTTCTTGGATCACCGCATTAAGAGTCAAGGTGCAGGCGAGGCTTAATATTGATCATCCC
CACATTATGTTTTTGTGGGTGCCGCGCATTTTGAAATCAACCCATGCCACCGTAGATTTA
AAAATTCAATATATTGCTACTGGTGATGTGAAGGTTTTAGGTAAGTTGCCATTGAATGAG
GCGTTCCTTCTCAGTTTTGGATGGGAACGCTCAATTAGGATGAAGGATGCGTATGCTAAG
AAAGGCCTTATGGTTTTTGCGCAACCTAGTGCCCCGGCAACACCTCCAGGAGCCCCTTTG
GGTCGCTGGATTCCTATGTGGGACGTTTGTCCCACACAGAAGATGCGGTATACCGAAGAT
GTCAAATCTTCGATGACAAAAGCTCAAGAGATGCGTGTGAAGACTATTTTGAATGAGCGT
ACCACACGCAGTCTCTTGCGTTCAGTCATGGCAAATGAATATACTTGCCGTGAACAAGTG
CCGAAGTTTTTGGGCCCAAGTGAGGTCCAATTGTCAGATGACTTGCCGGAGTTTCATGAT
TTTACTCTTGAAATGATCTCCGAAAGAAAACCCGAGAACATTCCCCTTAAGGGTGCTGTT
AAGGCCATTGAAATCCCCAATAATTTGGGAGTTGTTCAGTCTGGAAATGGTGTGTCAGGG
GATCGAGCCGTCTCTGCTTCAAAGAGGCAAGAACACAATACCCTGTAA

>Translation of ORF number 2 in reading frame 1 on the direct strand.
/function="cell to cell movement protein", 295aa
MSGFKTKAVVLSATDEESLLAEISGALSRAQMDMKTFRACVPIEMKNDKAFCFELCDETT
RSWITALRVKVQARLNIDHPHIMFLWVPRILKSTHATVDLKIQYIATGDVKVLGKLPLNE
AFLLSFGWERSIRMKDAYAKKGLMVFAQPSAPATPPGAPLGRWIPMWDVCPTQKMRYTED
VKSSMTKAQEMRVKTILNERTTRSLLRSVMANEYTCREQVPKFLGPSEVQLSDDLPEFHD
FTLEMISERKPENIPLKGAVKAIEIPNNLGVVQSGNGVSGDRAVSASKRQEHNTL*

>ORF number 3 in reading frame 2 on the direct strand extends from
base 1424 to base 2083.
ATGTCGAACAATGCTATTGAGATTAATGGGCAGTGGTATATACCACAATCAGGTCGTCAG
CCCAAGTCTGTAAATAATGCCCCCGTTATTCGGGGAAGACGGAAACCTACGTCCAGGTCG
CAAGCCTGGGCTCAAGGTAACCGTTCCCAGCCACCGGATAGGATGATGTTTGGCGCGATG
CGCTCTTCCCTACCCTCCTGGATTAGTACTCCAGGTGAACAGTGGCATGAAGTGGAAGGG
TTATCCTTTCCAGCTTCCTGGACAACAGGTTCCGTGGCTTCTGCCTCGATGCGCACTGAG
TTGGGGAAAATAAGACCCCTGCATGATTCCACGAAGGTGTATTCCGTGATGTATGGTTTT
ACCTGCAAATCTGATGGTTATGCCGGATTTGTGGAAGGGTTCGATGTGAACAATCCCACT
GGTCCGATTGCTCCTAATCGTGTCCGTGTGAAGGCGGGGAAGTATGCAGCTCGTCAGCTA
CGATGCCCCCCTGGTACCACAATTGCCGATTTGAAGGCCTCGTGGTCTTTTGTTTGGCAA
TTTGATGCCGCCCCAGCTACCGCCACTGTGAATCAAATCTCAGTGGTTGGTTTTTGGGTT
TCTACCACACCTTTGCCTGGTGTGAAGCCACCACCAGATTTTCTGGTGTGTGAAGAGTGA

>Translation of ORF number 3 in reading frame 2 on the direct strand.
/function="viral capsid protein" 219aa
MSNNAIEINGQWYIPQSGRQPKSVNNAPVIRGRRKPTSRSQAWAQGNRSQPPDRMMFGAM
RSSLPSWISTPGEQWHEVEGLSFPASWTTGSVASASMRTELGKIRPLHDSTKVYSVMYGF
TCKSDGYAGFVEGFDVNNPTGPIAPNRVRVKAGKYAARQLRCPPGTTIADLKASWSFVWQ
FDAAPATATVNQISVVGFWVSTTPLPGVKPPPDFLVCEE*
